# Metal–Solvent Complex Formation at the Surface of InP Colloidal Quantum Dots

**DOI:** 10.1021/jacs.4c03325

**Published:** 2024-04-26

**Authors:** Yun Hai, Kushagra Gahlot, Mark Tanchev, Suhas Mutalik, Eelco K. Tekelenburg, Jennifer Hong, Majid Ahmadi, Laura Piveteau, Maria Antonietta Loi, Loredana Protesescu

**Affiliations:** † Zernike Institute for Advanced Materials, University of Groningen, Nijenborgh 4, Groningen, 9747AG, The Netherlands; ‡ Institute of Chemistry and Chemical Engineering, École Polytechnique Fédérale de Lausanne, 1015 Lausanne, Switzerland

## Abstract

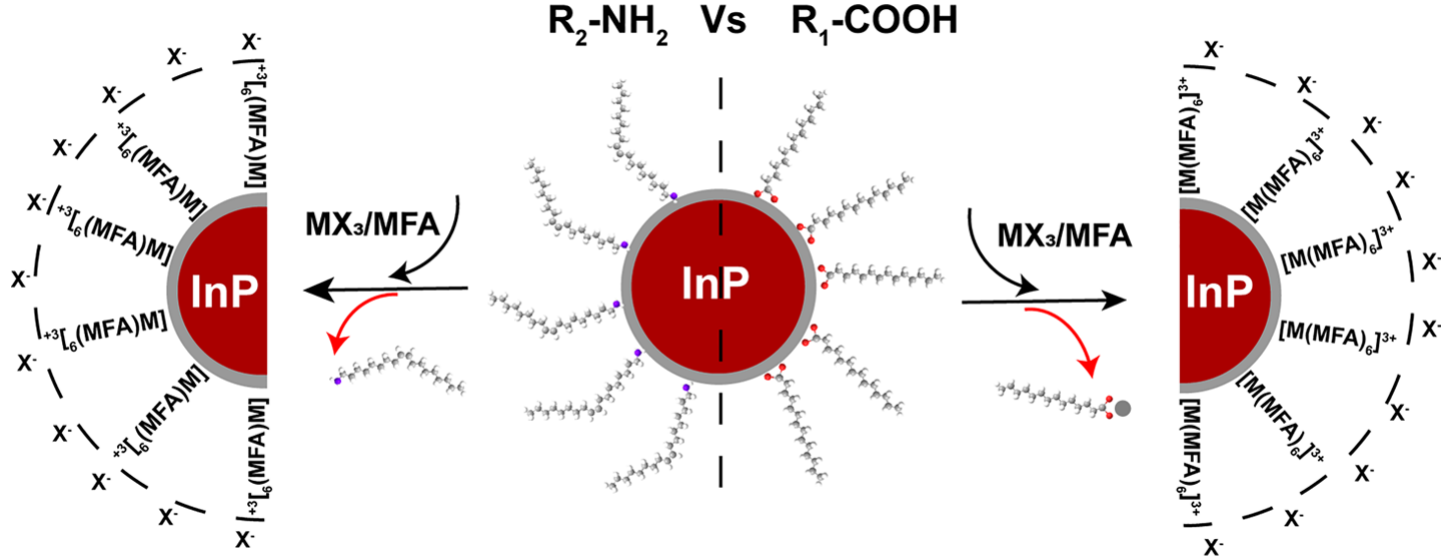

The surface chemistry of colloidal semiconductor nanocrystals (QDs) profoundly influences their physical and chemical attributes. The insulating organic shell ensuring colloidal stability impedes charge transfer, thus limiting optoelectronic applications. Exchanging these ligands with shorter inorganic ones enhances charge mobility and stability, which is pivotal for using these materials as active layers for LEDs, photodetectors, and transistors. Among those, InP QDs also serve as a model for surface chemistry investigations. This study focuses on group III metal salts as inorganic ligands for InP QDs. We explored the ligand exchange mechanism when metal halide, nitrate, and perchlorate salts of group III (Al, In Ga), common Lewis acids, are used as ligands for the conductive inks. Moreover, we compared the exchange mechanism for two starting model systems: InP QDs capped with myristate and oleylamine as X- and L-type native organic ligands, respectively. We found that all metal halide, nitrate, and perchlorate salts dissolved in polar solvents (such as *n*-methylformamide, dimethylformamide, dimethyl sulfoxide, H_2_O) with various polarity formed metal–solvent complex cations [M(Solvent)_6_]^3+^ (e.g., [Al(MFA)_6_]^3+^, [Ga(MFA)_6_]^3+^, [In(MFA)_6_]^3+^), which passivated the surface of InP QDs after the removal of the initial organic ligand. All metal halide capped InP/[M(Solvent)_6_]^3+^ QDs show excellent colloidal stability in polar solvents with high dielectric constant even after 6 months in concentrations up to 74 mg/mL. Our findings demonstrate the dominance of dissociation–complexation mechanisms in polar solvents, ensuring colloidal stability. This comprehensive understanding of InP QD surface chemistry paves the way for exploring more complex QD systems such as InAs and InSb QDs.

## Introduction

The surface chemistry of colloidal semiconductor nanocrystals (QDs) dominates their physical and chemical properties.^1−4^ As born, the QDs display an insulating organic shell critical during the nucleation and growth, ensuring the colloidal stability of the nanocrystals (NCs). However, at the same time, it inhibits charge transfer from one QD to another and hinders their implementation in optoelectronic devices. Exchanging those native organic ligands with shorter inorganic ones has been a strategy used for several decades already.^1^ Given this approach, surface traps can be passivated, the charge mobility can be increased, and the stability of the active layer for LEDs,^5^ photodetectors,^6^ transistors,^7^ and other optoelectronic devices is enhanced.^8^

While for the traditional II–VI and IV–VI QDs (e.g., CdS, CdSe, PbS),^9−15^ the advance in the surface chemistry study is remarkable, for the III–V QDs (e.g., InP, InAs, InSb) more opportunities to explore their surface opened with the development of high-quality QDs through optimized synthetic protocols.^16,17^ Because of their predominantly covalent character,^17−19^ III–V QDs exhibit distinctive surface chemistry properties. For instance, as synthesized III–V QDs (with organic ligands) are prone to oxidation, forming the oxide layer on the surface (e.g., P_*x*_O_*y*_,^20−26^ As_2_O_*x*_,^27,28^ Sb_2_O_*x*_,^29−31^ In_2_O_3_^22,32^) during the synthesis and postpurification steps. In this oxidation event, uncoordinated surface atoms, dislocations inducing In atom diffusion, and In–O–In bridges formed on the surface will develop as deep surface trap states, resulting in poor photoluminescence properties.^7,33−38^

Among the III-Vs, InP QDs have emerged as a prominent research focus, with extensive investigations contributing to our understanding of surface chemistry phenomena. Despite the considerable attention they have received compared to other III–V members, InP QDs continue to serve as a model system for exploring novel concepts in surface chemistry, as many intriguing questions remain unanswered. These include understanding the mechanisms underlying ligand exchange (LE),^39−41^ which is a powerful chemical strategy to remove the original long hydrocarbon ligands (e.g., oleylamine (OAm), oleic acid (OA), trioctylphosphine (TOP)) and substitute them with inorganic ligands.^41−45^ When novel ligands such as metal halide Lewis acids (AlX_3_, GaX_3_, InX_3_) are used in this process, their interaction with the solvent of choice (such as *n*-methylformamide (MFA), dimethylformamide (DMF), dimethyl sulfoxide (DMSO)) and their binding motifs are important for the comprehension of surface chemistry processes, improving the properties of InP QDs for various applications, and, with that knowledge, exploring the surface of more complex systems such as InSb or InAs QDs.

Pioneering studies by Talapin’s group resulted in the achievements of solution-based ligand exchange methods for InP QDs with metal-free inorganic ligands (S^2–^), which displace the organic ligands to form S^2–^-capped InP QDs, providing the first example of all-inorganic colloidal III–V NCs.^41^ Later, the same group reported that molecular metal chalcogenide complexes (MCCs: such as In_2_Se_4_^2–^, Sn_2_S_6_^4–^, Sn_2_Se_6_^4–^, Cu_7_S_4_^2–^) can also effectively replace the native organic ligands of InP QDs, greatly improving their charge transport with electron mobility for InP/In_2_Se_4_^2–^ at 3.35 cm^2^/(V s).^44^ Furthermore, oxoanions (e.g., PO_4_^3–^, MoO_4_^2–^) proved the capability to replace organic ligands and form a colloidally stable oxoanion-capped InP QD solution.^46^

Finally, metal halide salts are recognized as very accessible and highly efficient inorganic ligands for colloidal QDs. They can readily dissociate to small cations and counterions in polar solvents, passivating and stabilizing the colloidal NCs via the electrostatic effect.^29,39,45,47−50^ A variety of metal halides (MX_2_ (M = Pb, Cd, Zn, Fe; X = Cl, Br, I)) have been used to functionalize the surface of different colloidal QDs (e.g., IV–VI, II–VI) via ligand exchange reaction, with the ligand exchange QDs showing enhanced luminescence quantum efficiency and excellent colloidal stability.^45^ Those metal halide salts (ZnCl_2_, ZnBr_2_, and InCl_3_) acting as Z-type ligands demonstrate excellent passivation of the surface since they have been also employed to increase the quantum yields of InP QDs capped with organic ligands via a surface treatment strategy.^39^

However, metal halides are a vast class of ligands, which can be categorized as alkali metal halides (e.g., NaX, KX, CsX; X = Cl, Br, I), alkaline earth metal halides (e.g., MgX_2_, CaX_2_), transition metal halides (e.g., FeX_3_, ZnX_2_, CdX_2_), and post-transition metal halides (e.g., AlX_3_, GaX_3_, InX_3_, SnX_2_, PbX_2_).^51,52^ These different types of metal halides have different ionic and covalent nature and Lewis acidity. For example, alkali metal and alkali earth metal halides are more ionic than post-transition metal halides, and post-transition metal halides have stronger Lewis acidity than the other group of metal halides.^52^ Hence, owing to their different intrinsic natures, these prospective surface ligands will display distinct dissociation behaviors when dissolved in polar solvents, which can act as the Lewis base;^53−55^ therefore distinct binding species on the surface of the QDs and a potential difference in LE chemistry path will occur. Few literature reports mentioned the importance of the polar solvents used with such salts during the LE. For instance, the groups of Sargent,^48^ Murray,^56^ and Jeon^57^ explained that the polar solvents (such as DMF) could act as coordinating agents to co-stabilize QDs capped with inorganic ligands after the LE, and the Shirahata group^29^ reported that metal halides (InBr_3_) coordinated with DMF formed a DMF–InBr_3_ complex during the ligand exchange with InSb QDs.^29^

Moreover, the trivalent post-transition metal halides with Al^3+^, Ga^3+^, and In^3+^ as cations can be present in dimers (e*.*g., Al_2_Br_6_), can take coordination numbers from 4 to 6 depending on the halides, and have the potential to form four-, five-, and six-coordinate complexes with polar solvents, which may be cation like [Al(H_2_O)_6_]^3+^ or [Al(OSMe_2_)_6_]^3+^, neutral like the adducts of the halides, e.g. AlCl_3_(NMe_3_)_2_, or even ionic like SnF_6_^2–^.^58^ Therefore, it is necessary to understand which chemical processes these metal halides undergo in the solvent of choice and which species are transferred through LE on the surface of the QDs.

With this work, we aim to understand the InP QDs’ surface chemistry when such salts are used as ligands. We chose the group III metal halide, nitrate, and perchlorate salts (MX_3_: M = Al, Ga, In; X = Cl, Br, I, NO_3_, ClO_4_), known Lewis acids, as the inorganic ligands for InP QDs. We used the liquid-state NMR to investigate how the group III metal halide salts dissociate in polar solvents (such as MFA, DMF, DMSO, and H_2_O) and what potential species formed before and during LE. Since [M(Solvent)_6_]^3+^ complex cations were detected, we demonstrated that metal–solvent complexation dissociation is the main phenomenon. Moreover, we used those complexes to replace the original native ligands from InP QDs successfully. The solid-state NMR, Fourier-transform infrared (FTIR) spectroscopy, and zeta potential measurement were employed to elucidate the ligand mechanism and binding motif of [M(Solvent)_6_]^3+^ complex cations to InP QDs. For comparison, we chose representatives of two categories for the native organic ligands: the X-type ligand myristate (MAc, strongly bound, terminating the lattice) and the L-type ligand OAm (weaker bound, neutral donor).^59^ After LE, InP QDs capped with the [M(Solvent)_6_]^3+^ showed high colloidal stability with excellent optical properties even after six months of storage in polar solvents.

## Results and Discussions

**Figure 1:**
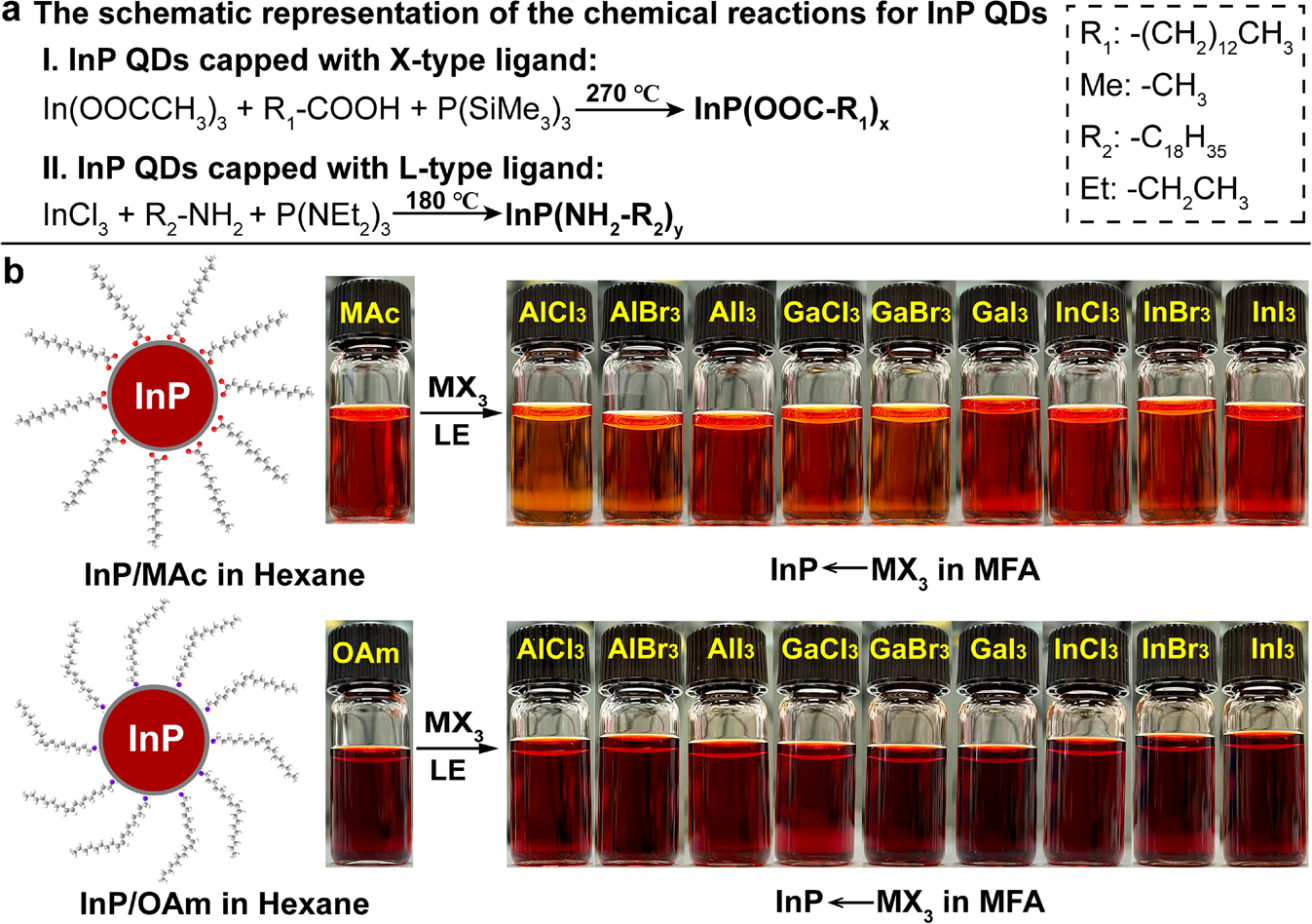
(a) The schematic representation of the chemical reactions for InP QDs capped with X- and L-type organic ligands. (b) Schematic illustration of InP/MAc QDs and InP/OAm QDs and representative photographs of the InP QDs before and after LE with metal halide salts for both X- and L-type native ligand, where M are Al, Ga, In; X are Cl, Br, I, and InP←MX_3_ means InP QDs after LE with MX_3_ salts.

We started by synthesizing InP QDs capped with MAc (the X-type ligand, bidentate chelating coordination with the surface indium atoms) and OAm (the L-type ligand, coordinates with indium atoms via the amine group donating two electrons) through the hot-injection method based on the modified protocols from Peng’s group^60^ and Hens’ group.^61^ The indium acetate/tris(trimethylsilyl)phosphine and indium chloride/tris(diethylamino)phosphine were chosen to be the indium/phosphorus precursors, respectively, as described in Figure 1a (the detailed synthesis procedures are presented in Supporting Information). We synthesized similar size (∼3 nm) InP QDs capped with both types of organic ligands, which are colloidally stable in the nonpolar solvents (e.g., hexane, toluene) as shown in Figure 1b.

We proceed to the typical LE procedure in which a biphase transfer method is employed to conduct the ligand exchange between organic ligands and metal halide salts. A fixed amount of InP QDs was dispersed in hexane, and the metal halide salts were dissolved in MFA. To estimate the needed concentration of inorganic ligands in MFA, we estimated the surface coverage of InP with initial organic ligands and calculated the molar ratios needed to fully replace them (Tables S1 and S2 in SI). The suspension of the QDs and the solution of the ligands were vigorously stirred at RT for 0.5 to 20 h until full migration of InP QDs from hexane to MFA (Figure S1) was observed. Then, the nonpolar phase was discarded, and the MFA phase was washed 4 times with hexane; finally, InP QDs capped inorganic ligands were precipitated from the MFA phase by adding acetone and toluene (1:4 vol). These inorganic ligand capped InP QDs can be redispersed in different polar solvents (such as MFA, DMF, and DMSO). All metal halide salts can successfully exchange both X- and L-type organic ligands, and the resulting InP/inorganic ligands QDs are remarkably colloidally stable in the polar solvents (Figure 1b).

**Figure 2:**
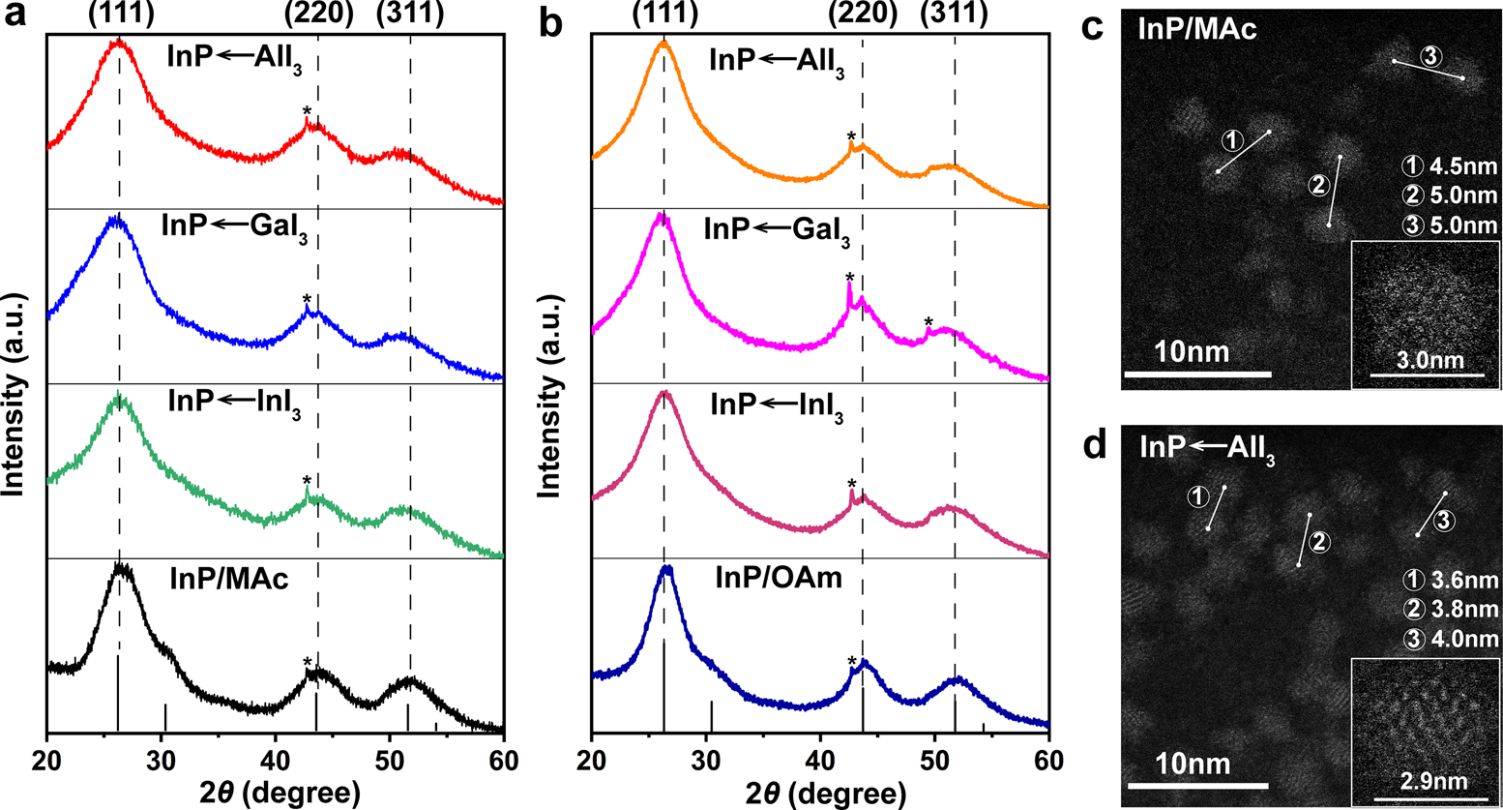
Powder X-ray diffraction (XRD) patterns of (a) InP/MAc QDs after LE with MI_3_ salts and (b) InP/OAm QDs after LE with MI_3_ salts. * marked peaks are from the blade of the XRD instrument. High-angle annular dark field scanning transmission electron microscopy (HAADF-STEM) image of (c) InP/MAc QDs and (d) InP/MAc QDs after LE with AlI_3_ salts The high-resolution STEM (HR-STEM) images are inserted in the lower right corner of each image, the size of InP/MAc QDs and InP/inorganic ligands QDs are around 3 and 2.9 nm respectively, and three different positions of interparticle distance were measured and marked with a white line.

All inorganic ligand capped InP QDs retain the same zinc-blend crystal structure as InP QDs capped with organic ligands (Figure 2a,b), while the morphology of the QDs before and after the LE is presented in the HAADF-STEM images (Figure 2c,d), where InP/MAc QDs and InP←AlI_3_ QDs exhibited the same shape and similar size (∼3 nm) (Figure S3b). Moreover, the interparticle distance of InP←AlI_3_ QDs (3.6 to 4 nm) is shorter than that of InP/MAc QDs (4.5 to 5 nm, marked in Figure 2c,d), which showed that the long-chain organic ligands were replaced by short inorganic metal halides.

**Figure 3:**
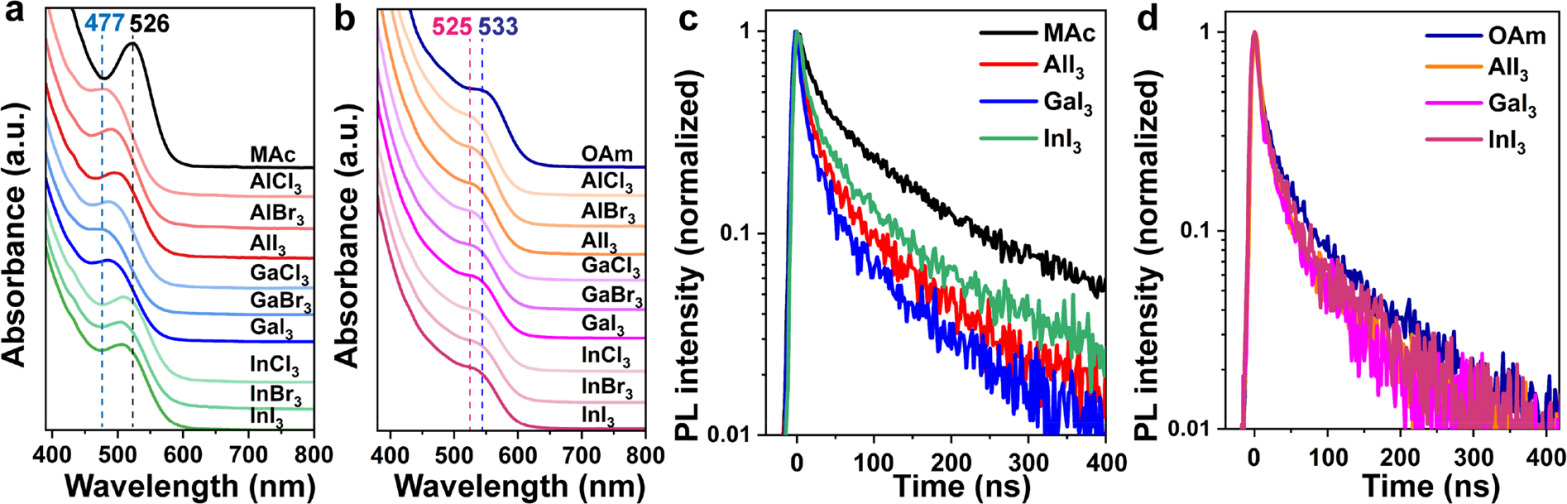
Absorbance spectra of (a) InP/MAc QDs after LE with MX_3_ salts, the first excitonic peak of InP/MAc QDs marked at 526 nm, and the maximum blue-shift to 477 nm displayed by the sample with the ligand GaBr_3_, and (b) InP/OAm QDs after LE with MX_3_ salts, the first excitonic peak of InP/OAm QDs marked at 533 nm, and all metal halide samples with a minimal blue-shift up to 8 nm. Time-resolved photoluminescence (TRPL) decays of (c) InP/MAc QDs after LE with MI_3_ salts and (d) InP/OAm QDs after LE with MI_3_ salts.

In Figure 3a and b, absorbance spectra show InP/inorganic ligand QDs obtained from both L- and X-type organic native ligands that preserved good first excitonic features, which indicates that ligand exchange reaction only disrupted the surface layer. It is pertinent to note that InP/inorganic ligand QDs synthesized with the MAc protocol exhibit notably larger blue-shifts, with a maximum shift of 49 nm, compared to those derived from the initially OAm-capped InP QDs, which demonstrates a maximum blue-shift of 8 nm. This disparity can be attributed to the stronger binding affinity of MAc, functioning as the X-type ligand, in contrast to OAm, serving as the L-type ligand, to the InP core, a phenomenon already reported.^39,62^ Consequently, MAc is more effective in displacing indium atoms from the surface with metal halide salts during the exchange process, leading to the observed differences in blue-shift magnitude (Figure S4). This conclusion can also be validated by the elemental analysis results (Figure S5), which showed that after ligand exchange, the In/P ratios in InP←AlI_3_ QDs and InP←GaI_3_ QDs declined more when the initial QDs were capped with MAc. Photoluminescence (PL) spectra show InP/inorganic ligand QDs have similar defect emission features with a minimal shift in the peak position (675 to 694 nm and 702 to 718 nm) for the MAc and OAm starting samples, respectively (Figure S6). Furthermore, an investigation into the impact of initial concentrations of metal iodides on the ultimate optical characteristics of InP/inorganic ligand QDs during the ligand exchange process is illustrated through the absorbance spectra depicted in Figure S7. The incremental variations in the initial concentration of MI_3_ yielded negligible differences; the resulting spectra exhibit comparable first excitonic features and closely aligned blue-shift tendencies.

Time-resolved photoluminescence (TRPL) measurements were performed to investigate the extent of electronic passivation of InP/inorganic ligand QDs (Figure 3c,d). TRPL traces for all InP QDs are adequately fitted with double-exponential functions. InP QDs after LE from MAc and OAm have faster decay times than original InP/MAc QDs and InP/OAm QDs. The exchange with metal halides induced the formation of surface traps or defects during the LE process, consequently leading to nonradiative channels of relaxation that diminish the PL lifetime of all InP inorganic capped QDs. Notably, it is imperative to underscore that the decay time of InP/inorganic ligand QDs obtained from the initial MAc-capped InP showed a much more pronounced reduction compared to those obtained from InP/OAm QDs, as depicted in Figure S8. This discrepancy can be attributed to the greater generation of surface traps during the ligand exchange process with MAc relative to OAm, owing to the differential binding strengths of MAc and OAm to the InP core. Additionally, among all metal halide salts examined, GaI_3_ induced the most substantial blue-shift and exhibited the swiftest decay time, while InI_3_ induced the smallest blue-shift and manifested the longest decay time. This variance likely arises from differences in Lewis acidity among the metal halide salts, thereby impacting the kinetics of the ligand exchange reaction.

The inorganic ligands can be identified on the surface of the InP QDs using Raman spectroscopy (Figure S9), following the characteristic vibrations of the metal iodides V_1_ (∼140 cm^–1^) and V_2_ (∼167 cm^–1^).^63−66^ Nevertheless, the LE is not 100% completed in all of our InP cases (both with initial X- and L-type ligands), as shown by the C–H vibrations recorded using FTIR spectroscopy (Figures S10 and S11). This observation was also highlighted by other reports,^39,43,67^ and it is intriguing since the chalcogenide ligands, for example, showed full exchange.^9^ Furthermore, given the anticipation that the metal halide ligands will function as Z-type ligands, one would predict a rapid and energetically favorable LE process, at least for the InP/OAm QDs system. In light of this, and acknowledging the introduction of Lewis acids as ligands in the presence of coordinating solvents, we proceeded with comprehensive investigations to understand the binding of inorganic ligands. Such elucidation holds implications for the eventual optoelectronic properties of the system.

**Figure 4:**
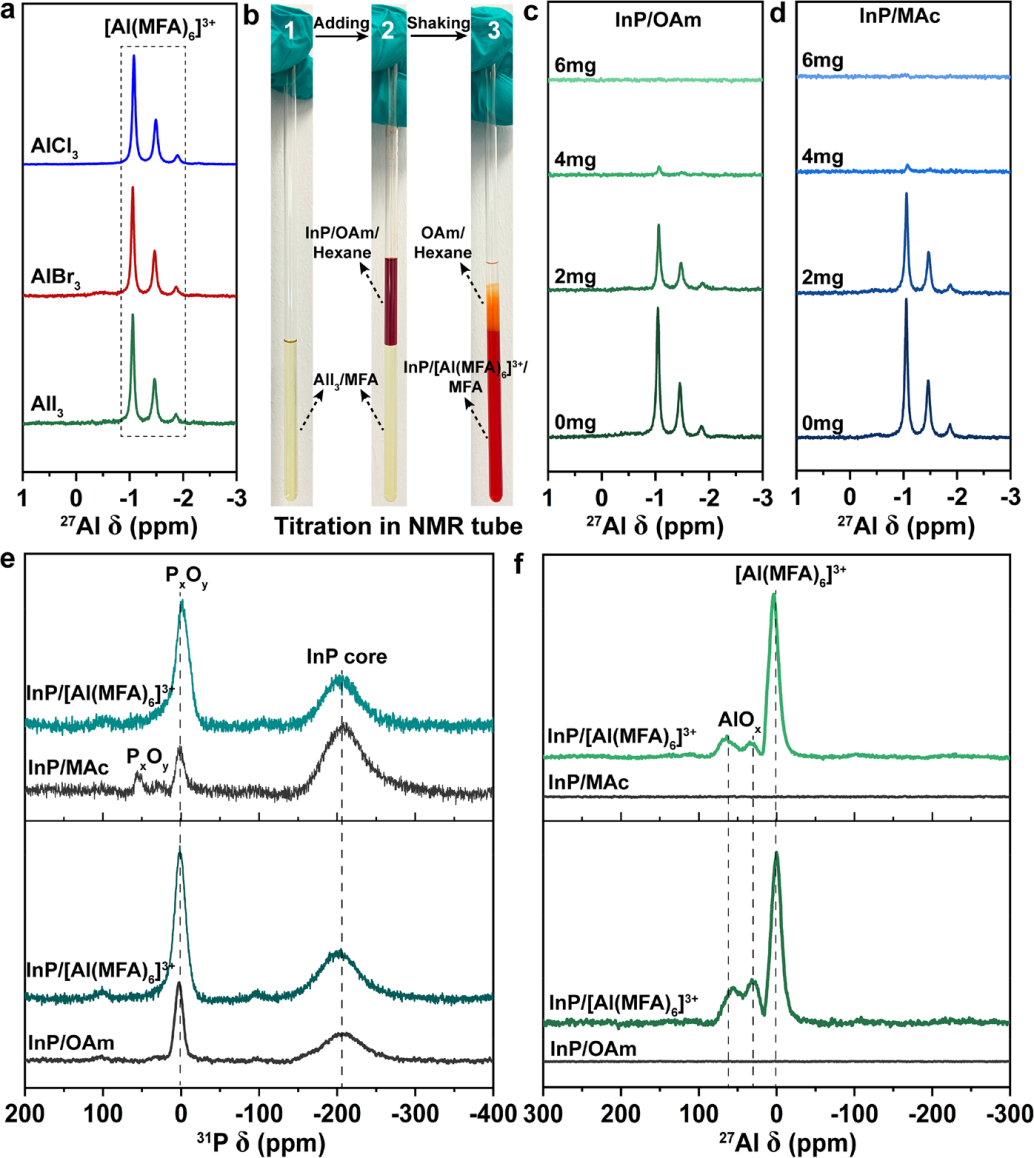
(a) Liquid-state ^27^Al NMR spectra of AlX_3_ salts in MFA. (b) The demonstration of InP/OAm QDs titration with AlI_3_/MFA in NMR tubes (1. AlI_3_/MFA; 2. adding InP/OAm/hexane; 3. phase transfer). Liquid-state ^27^Al NMR spectra of (c) InP/OAm QDs titration with AlI_3_/MFA and (d) InP/MAc QDs titration with AlI_3_/MFA. (e) Solid-state ^31^P MAS NMR spectra of InP QDs before and after LE with AlI_3_ salts at 500 MHz. (f) Solid-state ^27^Al MAS NMR spectra of InP QDs before and after LE with AlI_3_ salts at 900 MHz.

We first investigated the possible species when metal halide salts were dissolved in polar solvents before the ligand exchange.^68−72^ We used liquid-state NMR spectroscopy and Al halides as our model system because ^27^Al is a very sensitive nucleus, and the chemical shifts of various species are well studied and tabulated.^69,72,73^ All AlX_3_ salts in MFA were measured using ^27^Al NMR (Figure 4a), and they showed very similar signals with very close chemical shifts (−1.1 ppm to −1.9 ppm), which we assigned to the [Al(MFA)_6_]^3+^ complex cation,^68−70,72^ based on the following chemical equation:^69−71^
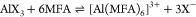
1To further prove the dissociation–complexation mechanism of AlX_3_ salts, various solvents such as DMSO, acetonitrile (ACN), H_2_O, MFA, ethylformamide (EFA), DMF, and formamide (FA), acting as Lewis bases, have been used to dissolve the AlX_3_ salts, and the formed Al species are shown in ^27^Al NMR spectra (Figure S12). The obtained ^27^Al chemical shifts (3.5 ppm to −3 ppm) indicated the formation of various Al species corresponding to AlX_3_ complexing with each solvent, resulting in the corresponding complexes [Al(DMSO)_6_]^3+^, [Al(H_2_O)_6_]^3+^, and [Al(DMF)_6_]^3+^, which are in good agreement with the previous studies on Lewis acid complexation.^69−72,74^ Thus, the complexation reaction of AlX_3_ salts with solvents acting as Lewis bases can be generalized as follows (eq 2):
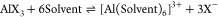
2Another possible self-ionization dissociation mechanism of metal halide salts in polar solvents has been reported in the literature yielding [MX_2_]^+^ and [MX_4_]^−^.^27,45^ However, the potential [AlX_2_]^+^ and [AlX_4_]^+^ species from AlX_3_ salts in MFA were not detected in liquid-state and solid-state ^27^Al NMR (Figure 4), which implies that, when AlX_3_ salts (and all group III metal halides) are used in combination with Lewis bases, the dissociation–complexation mechanism dominates and the self-ionization dissociation process is suppressed.

We continued with the titration experiments in the NMR tube to track the [Al(MFA)_6_]^3+^ complex cation during the ligand exchange. As shown in Figure 4b–d, when we gradually added the InP/MAc QDs and InP/OAm QDs to the AlI_3_/MFA phase, the signal intensity of the [Al(MFA)_6_]^3+^ complex decreased until it disappeared completely, indicating that the [Al(MFA)_6_]^3+^ complex cation coordinated to the surface of InP QDs. Additionally, no ^27^Al signals were detected when we measured ^27^Al NMR for InP/[Al(MFA)_6_]^3+^ QDs in MFA, showing that no free [Al(MFA)_6_]^3+^ ligands were left in the suspension (Figure S13).

In progressing beyond the examination of colloidal solutions to the investigation of dried powders of InP capped with organic and inorganic ligands, we conducted solid-state ^31^P and ^27^Al NMR, and we probed the oxide species and the [Al(MFA)_6_]^3+^ complexes on the surface of InP QDs before and after LE. In Figure 4e, the ^31^P NMR spectra for InP QDs before and after ligand exchange showed the chemical shift around −200 ppm assigned to the core of InP QDs, and the chemical shift around 0 ppm corresponded to the phosphorus oxide species (_*x*_P_*x*_O_*y*_) from the surface of InP QDs.^21,22,75^ Note that the postwashing, purification steps, and the LE procedures were conducted under air conditions. The ratio between the NMR signal for P_*x*_O_*y*_ (phosphates and polyphosphates)^7,20−26,33^ and the InP core increased after ligand exchange with AlI_3_, which indicated that the ligand exchange process further promoted the oxidization of the InP QDs. In ^27^Al NMR spectra (Figure 4f), after ligand exchange, InP←AlI_3_ QD samples showed three ^27^Al signals belonging to different Al species. The chemical shift at around 0 ppm is tentatively ascribed to the six-coordinated Al species corresponding to the [Al(MFA)_6_]^3+^ complex cation, and based on their chemical shifts, the signals at 31 and 60 ppm may be attributed to five-coordinated and four-coordinated Al species, which are mainly from the Al coordinating with the surface oxidation layer of InP QDs.^72,73,76−80^

**Figure 5:**
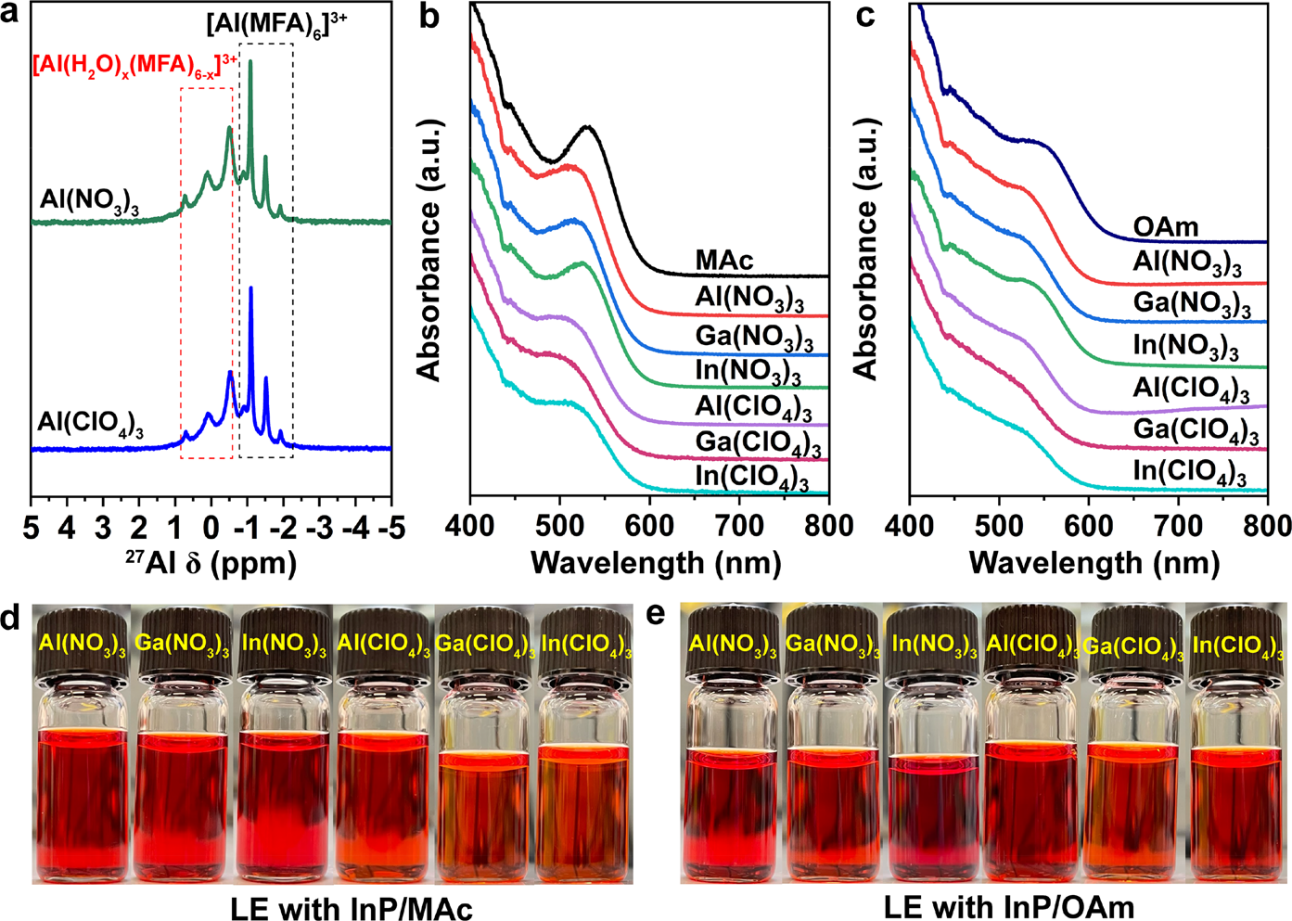
(a) Liquid-state ^27^Al NMR spectra of Al(NO_3_)_3_ and Al(ClO_4_)_3_ salts in MFA. Absorbance spectra of (b) InP/MAc QDs and (c) InP/OAm QDs after LE with Al(NO_3_)_3_ and Al(ClO_4_)_3_ salts. InP QDs samples dispersed in MFA in vials of (d) InP/MAc QDs and (e) InP/OAm QDs after LE with Al(NO_3_)_3_ and Al(ClO_4_)_3_ salts.

To probe the role of the halide in the complexation chemistry of the group III metal salts and their potential as the inorganic ligands of InP QDs, we employed the metal nitrate and perchlorate salts of the same M as inorganic ligands with InP/MAc QDs and InP/OAm QDs. We detected the [Al(MFA)_6_]^3+^ complex species when Al nitrate and perchlorate were dissolved in MFA (Figure 5a). In addition, we also obtained signals belonging to [Al(H_2_O)_*x*_(MFA)_6–*x*_]^3+^ complex species due to the hydrated nature of Al(NO_3_)_3_ and Al(ClO_4_)_3_ precursors. We confirmed those mixed solvent complexes by adding the corresponding amount of H_2_O to an AlI_3_/MFA solution and detecting the same [Al(H_2_O)_*x*_(MFA)_6–*x*_]^3+^ complex species (Figure S14). Therefore, the metal nitrate and perchlorate salts follow the same dissociation–complexation mechanism represented in eq 1 when replacing the halides with nitrate or perchlorate anions. These metal nitrate and perchlorate salts dissolved in MFA demonstrated successful LE, maintaining good excitonic features of InP QDs and prolonged colloidal stability in the same solvent (Figure 5b–e).

**Figure 6:**
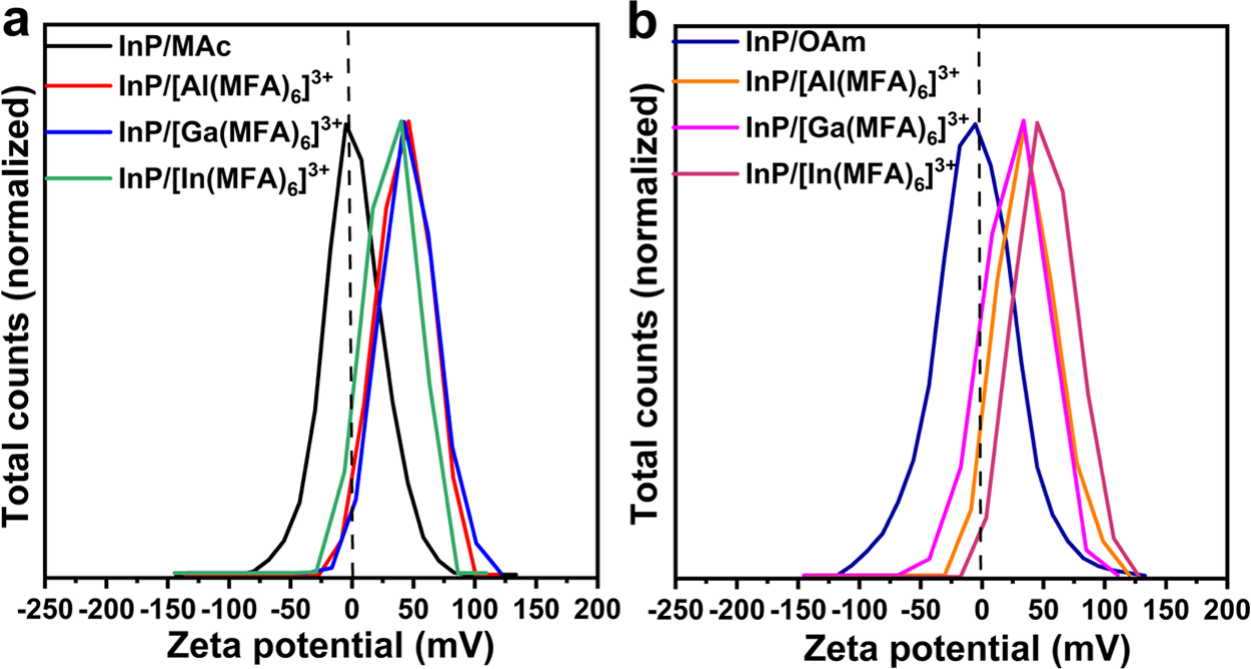
Zeta-potential measurements for (a) InP/MAc QDs after LE with MI_3_ salts and (b) InP/OAm QDs after LE with MI_3_ salts.

Since the complex that we argue to passivate the surface of InP QDs should be positively charged, we performed zeta potential measurements for InP QDs before and after LE, as presented in Figure 6. For the original InP/MAc QDs and InP/OAm QDs, we measured nearly zero zeta potential, which means they have neutral surfaces, making them colloidally stable in nonpolar solvents by steric effects. After LE with metal iodides, all InP/[M(MFA)_6_]^3+^ QDs resulting from InP/MAc QDs and InP/OAm QDs showed positive zeta potentials (the values around 25–50 mV are plotted in Figure S15), confirming our previous results. We obtained similar positive surfaces for InP QDs after LE with Al(NO_3_)_3_ and Al(ClO_4_)_3_ (Figure S16).

To generalize, owing to their strong Lewis acidic properties, salts of group III metals undergo dissociation by interacting with solvents acting as Lewis bases, resulting in the formation of metal–solvent complex cations ([M(Solvent)_6_]^3+^), as depicted in eq 3 (M = Al, Ga, In; X = Cl, Br, I, NO_3_, ClO_4_, Solvent = Lewis bases). The halide component (X^–^) of the initial salt compensates for any surface charge imbalance and stabilizes the colloidal QDs through diffusion and electrostatic effects in the polar solvent. The presence of halides in a low ratio compared to the M (M:X = from 5.3 to 12) was confirmed using ICP-MS (Table S3).
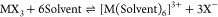
3

**Figure 7:**
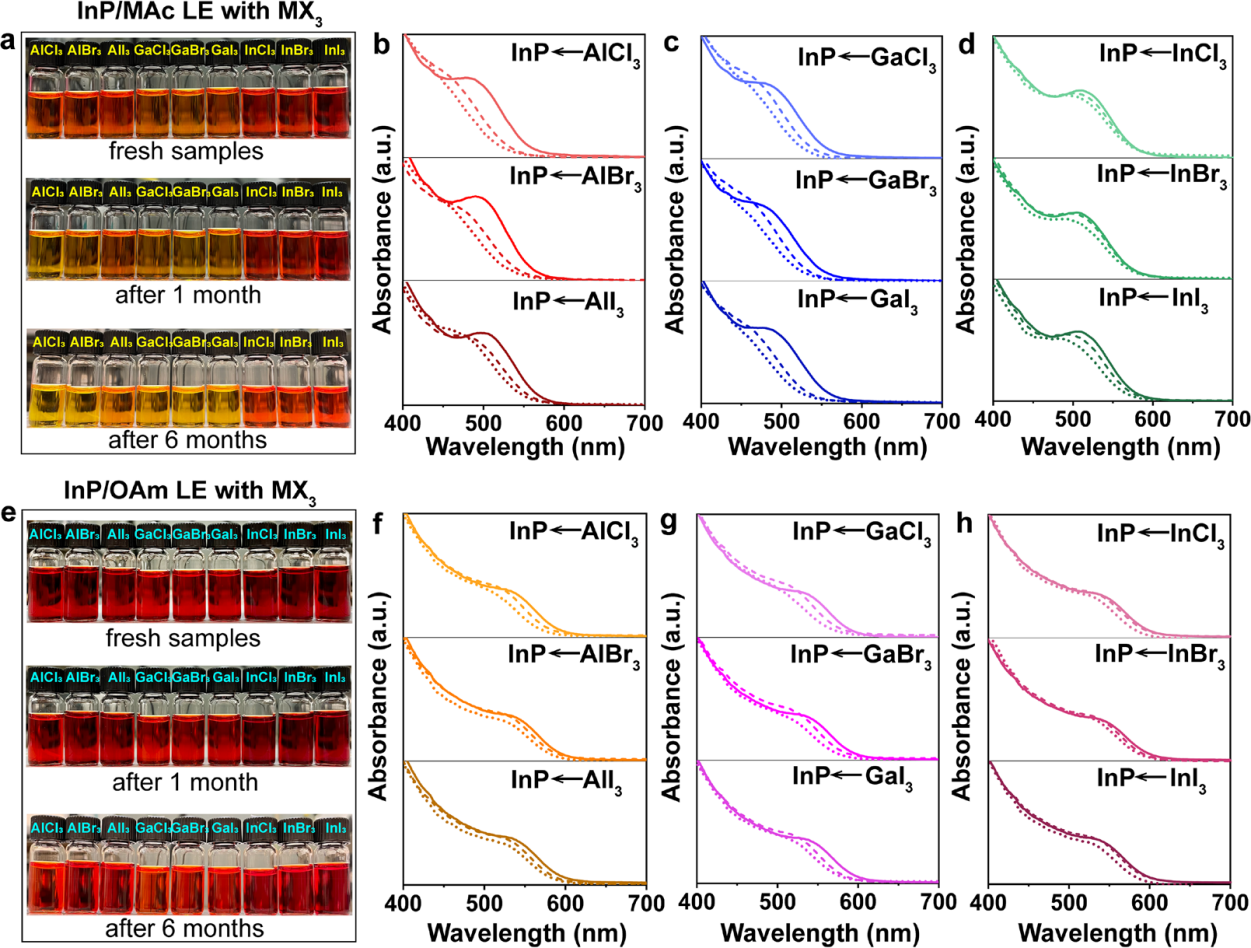
Photographs of InP QDs samples in MFA after LE with MX_3_ salts when fresh, after 1 month, and after 6 months (a) from the InP/MAc QDs side and (e) from the InP/OAm QDs side. (b–d) Absorbance spectra of InP/MAc QDs LE with MX_3_ salts collected over time: (b) AlX_3_, (c) GaX_3_, and (d) InX_3_. (f–h) Absorbance spectra of InP/OAm QDs LE with MX_3_ salts collected over time: (f) AlX_3_, (g) GaX_3_, and (h) InX_3_. Solid line: fresh samples; dashed line: after 1 month; dotted line: after 6 months.

Lastly, we evaluated the colloidal stability of InP/inorganic ligand QDs in polar solvents, and we probed the colloidal stability and optical properties of InP capped with all tested ligands ([M(MFA)_6_]^3+^) after 6 months. The photographs depicting the state of all InP/[M(MFA)_6_]^3+^ samples in vials are shown in Figure 7a,e. The absorption spectra revealed that the inorganic capped InP QDs that were obtained via LE from the InP/OAm QDs present longer optical stability since the excitonic peak shows minor changes in all metal halide cases over the 6 months of study (Figure 7e–h). In contrast, the inks obtained from InP/MAc QDs continued to etch and degrade, showing a considerable blue-shift in time (Figure 7b–d, f–h). This occurrence can be explained by two plausible factors: first, the InP quantum dots may undergo oxidation when stored under ambient air conditions; second, an excess of metal halide salts may continuously etch the InP QDs, resulting in a size reduction. Moreover, it is evident that even after 6 months the inorganic capped InP QDs remained colloidally stable. This observation suggests that the metal complexes exhibit robust binding with the InP core, enabling long-term colloidal stability in polar solvents through electrostatic effects.

The colloidal stability of InP/[M(MFA)_6_]^3+^ QDs was also investigated in other polar solvents with various dielectric constants from 15 to 200. We dispersed the InP/[In(MFA)_6_]^3+^ QDs in 12 different polar solvents (Figure S17), and we can conclude that the polar solvents with higher dielectric constants such as *n*-methylacetamide (MAA), MFA, and FA can maintain the colloidal stability of InP/[In(MFA)_6_]^3+^ QDs at high concentrations (∼5 mg/mL) On the contrary, in less polar solvents, the InP/[In(MFA)_6_]^3+^ QDs can only be colloidally stable at low concentrations (∼1.5 mg/mL), which is in good agreement with the weaker ability of these solvents to solvate ions and stabilize charges.^1^ In addition, the InP/[In(MFA)_6_]^3+^ QDs were made for the colloidally stable inks, and their concentration can reach 73.7 mg/mL, which is still well monodispersed and stable in MFA, as shown in Figure S18.

This exploration of the stability of InP/[M(MFA)_6_]^3+^ QDs in various polar solvents was essential, as it offers fundamental insights crucial for advancing research in surface modifications and film engineering. This exploration is essential for selecting an appropriate solvent based on considerations such as boiling point, polarity, viscosity, and other physicochemical properties.

## Conclusions

In summary, we investigated the surface chemistry of InP QDs utilizing metal halide, nitrate, and perchlorate salts as inorganic ligands. Employing a combination of liquid-state and solid-state NMR, FTIR, and Raman spectroscopies combined with optical property characterizations, we investigated the dissociation behavior of group III metal salts in polar solvents and their subsequent interaction with InP QDs. The successful substitution of native ligands with metal complexes led to notable colloidal stability and good optical properties of the InP QDs, even upon extended storage periods. Moreover, our investigation revealed the role of the anion component in the complexation chemistry of metal salts, shedding light on their potential as inorganic ligands for InP QDs. Through a comprehensive analysis involving spectroscopic techniques and zeta potential measurements, we elucidate the mechanisms governing the ligand exchange process and subsequent surface passivation of InP/[M(Solvent)_6_]^3+^ QDs. In essence, our findings emphasize the significance of understanding the intricate interplay between metal halide nitrate and perchlorate salts, polar solvents, and InP QDs in surface chemistry phenomena. This knowledge not only enriches our comprehension of fundamental surface processes but also opens avenues for tailoring the properties of III–V QDs to suit a diverse range of applications in optoelectronics and beyond.

## Supporting Information Available

Synthesis protocols, ligand exchange procedures, the theoretical calculation of the size and surface of InP QDs, and additional figures and tables such as Raman spectra, ICP-MS, and FTIR spectra (PDF)

## References

[ref1] Boles M. A.; Ling D.; Hyeon T.; Talapin D. V. The Surface Science of Nanocrystals. Nat. Mater. 2016, 15 (2), 141–153. 10.1038/nmat4526. 26796733

[ref2] Eagle F. W.; Rivera-Maldonado R. A.; Cossairt B. M. Surface Chemistry of Metal Phosphide Nanocrystals. Annu. Rev. Mater. Res. 2021, 51 (1), 541–564. 10.1146/annurev-matsci-080819-011036.

[ref3] Click S. M.; Rosenthal S. J. Synthesis, Surface Chemistry, and Fluorescent Properties of InP Quantum Dots. Chem. Mater. 2023, 35 (3), 822–836. 10.1021/acs.chemmater.2c03074.

[ref4] Hartley C. L.; Kessler M. L.; Dempsey J. L. Molecular-Level Insight into Semiconductor Nanocrystal Surfaces. J. Am. Chem. Soc. 2021, 143 (3), 1251–1266. 10.1021/jacs.0c10658. 33442974

[ref5] Cui Z.; Yang D.; Qin S.; Wen Z.; He H.; Mei S.; Zhang W.; Xing G.; Liang C.; Guo R. Advances, Challenges, and Perspectives for Heavy Metal Free Blue Emitting Indium Phosphide Quantum Dot Light Emitting Diodes. Adv. Opt. Mater. 2023, 11 (4), 1–24. 10.1002/adom.202202036.

[ref6] Chen B.; Li D.; Wang F. InP Quantum Dots: Synthesis and Lighting Applications. Small 2020, 16 (32), 1–20. 10.1002/smll.202002454. 32613755

[ref7] Almeida G.; Ubbink R. F.; Stam M.; Du Fossé I.; Houtepen A. J. InP Colloidal Quantum Dots for Visible and Near-Infrared Photonics. Nat. Rev. Mater. 2023, 8, 742–758. 10.1038/s41578-023-00596-4.

[ref8] Zhang J.; Zhang S.; Zhang Y.; Al Hartomy O. A.; Wageh S.; Al Sehemi A. G.; Hao Y.; Gao L.; Wang H.; Zhang H. Colloidal Quantum Dots: Synthesis, Composition, Structure, and Emerging Optoelectronic Applications. Laser Photonics Rev. 2023, 17 (3), 1–50. 10.1002/lpor.202200551.

[ref9] Protesescu L.; Nachtegaal M.; Voznyy O.; Borovinskaya O.; Rossini A. J.; Emsley L.; Copéret C.; Günther D.; Sargent E. H.; Kovalenko M. V. Atomistic Description of Thiostannate-Capped CdSe Nanocrystals: Retention of Four-Coordinate SnS_4_ Motif and Preservation of Cd-Rich Stoichiometry. J. Am. Chem. Soc. 2015, 137 (5), 1862–1874. 10.1021/ja510862c. 25597625 PMC4525771

[ref10] Zhang J.; Zhang H.; Cao W.; Pang Z.; Li J.; Shu Y.; Zhu C.; Kong X.; Wang L.; Peng X. Identification of Facet-Dependent Coordination Structures of Carboxylate Ligands on CdSe Nanocrystals. J. Am. Chem. Soc. 2019, 141 (39), 15675–15683. 10.1021/jacs.9b07836. 31503473

[ref11] Zhou Y.; Buhro W. E. Reversible Exchange of L-Type and Bound-Ion-Pair X-Type Ligation on Cadmium Selenide Quantum Belts. J. Am. Chem. Soc. 2017, 139 (37), 12887–12890. 10.1021/jacs.7b05167. 28876924

[ref12] Lee W. S.; Kang Y. G.; Woo H. K.; Ahn J.; Kim H.; Kim D.; Jeon S.; Han M. J.; Choi J.-H.; Oh S. J. Designing High-Performance CdSe Nanocrystal Thin-Film Transistors Based on Solution Process of Simultaneous Ligand Exchange, Trap Passivation, and Doping. Chem. Mater. 2019, 31 (22), 9389–9399. 10.1021/acs.chemmater.9b02965.

[ref13] Greaney M. J.; Couderc E.; Zhao J.; Nail B. A.; Mecklenburg M.; Thornbury W.; Osterloh F. E.; Bradforth S. E.; Brutchey R. L. Controlling the Trap State Landscape of Colloidal CdSe Nanocrystals with Cadmium Halide Ligands. Chem. Mater. 2015, 27 (3), 744–756. 10.1021/cm503529j.

[ref14] Hughes B. K.; Ruddy D. A.; Blackburn J. L.; Smith D. K.; Bergren M. R.; Nozik A. J.; Johnson J. C.; Beard M. C. Control of PbSe Quantum Dot Surface Chemistry and Photophysics Using an Alkylselenide Ligand. ACS Nano 2012, 6 (6), 5498–5506. 10.1021/nn301405j. 22571723

[ref15] Anderson N. C.; Hendricks M. P.; Choi J. J.; Owen J. S. Ligand Exchange and the Stoichiometry of Metal Chalcogenide Nanocrystals: Spectroscopic Observation of Facile Metal-Carboxylate Displacement and Binding. J. Am. Chem. Soc. 2013, 135 (49), 18536–18548. 10.1021/ja4086758. 24199846 PMC4102385

[ref16] Kim T.; Shin D.; Kim M.; Kim H.; Cho E.; Choi M.; Kim J.; Jang E.; Jeong S. Development of Group III-V Colloidal Quantum Dots for Optoelectronic Applications. ACS Energy Lett. 2023, 8 (1), 447–456. 10.1021/acsenergylett.2c02489.

[ref17] Zhao Q.; Kulik H. J. Electronic Structure Origins of Surface-Dependent Growth in III-V Quantum Dots. Chem. Mater. 2018, 30 (20), 7154–7165. 10.1021/acs.chemmater.8b03125.

[ref18] Kim Y.; Chang J. H.; Choi H.; Kim Y.-H.; Bae W. K.; Jeong S. III-V Colloidal Nanocrystals: Control of Covalent Surfaces. Chem. Sci. 2020, 11 (4), 913–922. 10.1039/C9SC04290C. PMC8145357 34084346

[ref19] R. Heath J.; Shiang J. J. Covalency in Semiconductor Quantum Dots. Chem. Soc. Rev. 1998, 27 (1), 65–71. 10.1039/a827065z.

[ref20] Cros-Gagneux A.; Delpech F.; Nayral C.; Cornejo A.; Coppel Y.; Chaudret B. Surface Chemistry of InP Quantum Dots: A Comprehensive Study. J. Am. Chem. Soc. 2010, 132 (51), 18147–18157. 10.1021/ja104673y. 21126088

[ref21] Tomaselli M.; Yarger J. L.; Bruchez M.; Havlin R. H.; Degraw D.; Pines A.; Alivisatos A. P. NMR Study of InP Quantum Dots: Surface Structure and Size Effects. J. Chem. Phys. 1999, 110 (18), 8861–8864. 10.1063/1.478858.

[ref22] Virieux H.; Le Troedec M.; Cros-Gagneux A.; Ojo W.-S.; Delpech F.; Nayral C.; Martinez H.; Chaudret B. InP/ZnS Nanocrystals: Coupling NMR and XPS for Fine Surface and Interface Description. J. Am. Chem. Soc. 2012, 134 (48), 19701–19708. 10.1021/ja307124m. 23131073

[ref23] Mandala V. S.; Loh D. M.; Shepard S. M.; Geeson M. B.; Sergeyev I. V.; Nocera D. G.; Cummins C. C.; Hong M. Bacterial Phosphate Granules Contain Cyclic Polyphosphates: Evidence from (31)P Solid-State NMR. J. Am. Chem. Soc. 2020, 142 (43), 18407–18421. 10.1021/jacs.0c06335. 33075224 PMC7755298

[ref24] Sannigrahi P.; Ingall E. Polyphosphates as A Source of Enhanced P Fluxes in Marine Sediments Overlain by Anoxic Waters: Evidence from ^31^P NMR. Geochem. Trans. 2005, 6 (3), 52–59. 10.1186/1467-4866-6-52. 35412770 PMC1475792

[ref25] Jahromi S.; Gabrielse W.; Braam A. Effect of Melamine Polyphosphate on Thermal Degradation of Polyamides: A Combined X-ray Diffraction and Solid-State NMR Study. Polymer 2003, 44, 25–37. 10.1016/S0032-3861(02)00686-9.

[ref26] Wiench J. W.; Tischendorf B.; Otaigbe J. U.; Pruski M. Structure of Zinc Polyphosphate Glasses Studied by Two-Dimensional Solid and Liquid State NMR. J. Mol. Struct. 2002, 602, 145–157. 10.1016/S0022-2860(01)00769-4.

[ref27] Sun B.; Najarian A. M.; Sagar L. K.; Biondi M.; Choi M. J.; Li X.; Levina L.; Baek S. W.; Zheng C.; Lee S.; Kirmani A. R.; Sabatini R.; Abed J.; Liu M.; Vafaie M.; Li P.; Richter L. J.; Voznyy O.; Chekini M.; Lu Z. H.; García De Arquer F. P.; Sargent E. H. Fast Near Infrared Photodetection Using III-V Colloidal Quantum Dots. Adv. Mater. 2022, 34 (33), 1–9. 10.1002/adma.202203039. 35767306

[ref28] Song J. H.; Choi H.; Pham H. T.; Jeong S. Energy Level Tuned Indium Arsenide Colloidal Quantum Dot Films for Efficient Photovoltaics. Nat. Commun. 2018, 9 (1), 1–9. 10.1038/s41467-018-06399-4. 30323251 PMC6189201

[ref29] Chatterjee S.; Nemoto K.; Ghosh B.; Sun H.-T.; Shirahata N. Solution-Processed InSb Quantum Dot Photodiodes for Short-Wave Infrared Sensing. ACS Appl. Nano Mater. 2023, 6 (17), 15540–15550. 10.1021/acsanm.3c02221.

[ref30] Kim S.-W.; Sujith S.; Lee B. Y. InAsxSb1-x Alloy Nanocrystals for Use in the Near Infrared. Chem. Commun. 2006, (46), 4811–4813. 10.1039/B611099A. 17345737

[ref31] Seo H.; Eun H. J.; Lee A. Y.; Lee H. K.; Kim J. H.; Kim S. W. Colloidal InSb Quantum Dots for 1500 nm SWIR Photodetector with Antioxidation of Surface. Adv. Sci. 2024, 11 (4), 1–10. 10.1002/advs.202306439. PMC10811490 38036427

[ref32] Zhang Y.; Xia P.; Rehl B.; Parmar D. H.; Choi D.; Imran M.; Chen Y.; Liu Y.; Vafaie M.; Li C.; Atan O.; Pina J. M.; Paritmongkol W.; Levina L.; Voznyy O.; Hoogland S.; Sargent E. H. Dicarboxylic Acid Assisted Surface Oxide Removal and Passivation of Indium Antimonide Colloidal Quantum Dots for Short Wave Infrared Photodetectors. Angew. Chem. 2024, 63, 1–9. 10.1002/anie.202316733. 38170453

[ref33] Ubbink R. F.; Almeida G.; Iziyi H.; Du Fossé I.; Verkleij R.; Ganapathy S.; Van Eck E. R. H.; Houtepen A. J. A Water-Free In Situ HF Treatment for Ultrabright InP Quantum Dots. Chem. Mater. 2022, 34 (22), 10093–10103. 10.1021/acs.chemmater.2c02800. 36439318 PMC9686131

[ref34] Baek H.; Kang S.; Heo J.; Choi S.; Kim R.; Kim K.; Ahn N.; Yoon Y.-G.; Lee T.; Chang J. B.; Lee K. S.; Park Y.-G.; Park J. Insights into structural defect formation in individual InP/ZnSe/ZnS quantum dots under UV oxidation. Nat. Commun. 2024, 15 ( (1), ), 10.1038/s41467-024-45944-2. PMC10891109 38396037

[ref35] Duan X.; Ma J.; Zhang W.; Liu P.; Liu H.; Hao J.; Wang K.; Samuelson L.; Sun X. W. Study of the Interfacial Oxidation of InP Quantum Dots Synthesized from Tris(dimethylamino)phosphine. ACS Appl. Mater. Interfaces 2023, 15 (1), 1619–1628. 10.1021/acsami.2c20138. 36574641

[ref36] Pu Y. C.; Fan H. C.; Chang J. C.; Chen Y. H.; Tseng S. W. Effects of Interfacial Oxidative Layer Removal on Charge Carrier Recombination Dynamics in InP/ZnSe(x)S(1-x) Core/Shell Quantum Dots. J. Phys. Chem. Lett. 2021, 12 (30), 7194–7200. 10.1021/acs.jpclett.1c02125. 34309384

[ref37] Zhang X.; Pham T. A.; Ogitsu T.; Wood B. C.; Ptasinska S. Modulation of Surface Bonding Topology: Oxygen Bridges on OH-Terminated InP (001). J. Phys. Chem. C 2020, 124 (5), 3196–3203. 10.1021/acs.jpcc.9b11548.

[ref38] Wood B. C.; Ogitsu T.; Schwegler E. Local structural models of complex oxygen- and hydroxyl-rich GaP/InP(001) surfaces. J. Chem. Phys. 2012, 136 (6), 064705 10.1063/1.3682768. 22360213

[ref39] Calvin J. J.; Swabeck J. K.; Sedlak A. B.; Kim Y.; Jang E.; Alivisatos A. P. Thermodynamic Investigation of Increased Luminescence in Indium Phosphide Quantum Dots by Treatment with Metal Halide Salts. J. Am. Chem. Soc. 2020, 142 (44), 18897–18906. 10.1021/jacs.0c08954. 33095575

[ref40] Ritchhart A.; Cossairt B. M. Quantifying Ligand Exchange on InP Using an Atomically Precise Cluster Platform. Inorg. Chem. 2019, 58 (4), 2840–2847. 10.1021/acs.inorgchem.8b03524. 30714365

[ref41] Nag A.; Kovalenko M. V.; Lee J.-S.; Liu W.; Spokoyny B.; Talapin D. V. Metal-free Inorganic Ligands for Colloidal Nanocrystals: S^2--^, HS^–2^, Se^2--^, HSe^–2^, Te^2--^, HTe^–2^, TeS_3_^2--^, OH-, and NH^2--^ as Surface Ligands. J. Am. Chem. Soc. 2011, 133 (27), 10612–10620. 10.1021/ja2029415. 21682249

[ref42] Choi M.-J.; Sagar L. K.; Sun B.; Biondi M.; Lee S.; Najjariyan A. M.; Levina L.; García De Arquer F. P.; Sargent E. H. Ligand Exchange at a Covalent Surface Enables Balanced Stoichiometry in III-V Colloidal Quantum Dots. Nano Lett. 2021, 21 (14), 6057–6063. 10.1021/acs.nanolett.1c01286. 34250796

[ref43] Leemans J.; Dümbgen K. C.; Minjauw M. M.; Zhao Q.; Vantomme A.; Infante I.; Detavernier C.; Hens Z. Acid-Base Mediated Ligand Exchange on Near-Infrared Absorbing, Indium-Based III-V Colloidal Quantum Dots. J. Am. Chem. Soc. 2021, 143 (11), 4290–4301. 10.1021/jacs.0c12871. 33710882

[ref44] Liu W.; Lee J.-S.; Talapin D. V. III-V Nanocrystals Capped with Molecular Metal Chalcogenide Ligands: High Electron Mobility and Ambipolar Photoresponse. J. Am. Chem. Soc. 2013, 135 (4), 1349–1357. 10.1021/ja308200f. 23267673

[ref45] Dirin D. N.; Dreyfuss S.; Bodnarchuk M. I.; Nedelcu G.; Papagiorgis P.; Itskos G.; Kovalenko M. V. Lead Halide Perovskites and Other Metal Halide Complexes As Inorganic Capping Ligands for Colloidal Nanocrystals. J. Am. Chem. Soc. 2014, 136 (18), 6550–6553. 10.1021/ja5006288. 24746226 PMC4524702

[ref46] Huang J.; Liu W.; Dolzhnikov D. S.; Protesescu L.; Kovalenko M. V.; Koo B.; Chattopadhyay S.; Shenchenko E. V.; Talapin D. V. Surface Functionalization of Semiconductor and Oxide Nanocrystals with Small Inorganic Oxoanions (PO_4_^3--^, MoO_4_^2--^) and Polyoxometalate Ligands. ACS Nano 2014, 8 (9), 9388–9402. 10.1021/nn503458y. 25181260

[ref47] Ghosh S.; Manna L. The Many “Facets” of Halide Ions in the Chemistry of Colloidal Inorganic Nanocrystals. Chem. Rev. 2018, 118 (16), 7804–7864. 10.1021/acs.chemrev.8b00158. 30062881 PMC6107855

[ref48] Sun B.; Najarian A. M.; Sagar L. K.; Biondi M.; Choi M. J.; Li X.; Levina L.; Baek S. W.; Zheng C.; Lee S.; Kirmani A. R.; Sabatini R.; Abed J.; Liu M.; Vafaie M.; Li P.; Richter L. J.; Voznyy O.; Chekini M.; Lu Z. H.; García De Arquer F. P.; Sargent E. H. Fast Near Infrared Photodetection Using III-V Colloidal Quantum Dots. Adv. Mater. 2022, 34 (33), 1–8. 10.1002/adma.202203039. 35767306

[ref49] Xia P.; Sun B.; Biondi M.; Xu J.; Atan O.; Imran M.; Hassan Y.; Liu Y.; Pina J. M.; Najarian A. M.; Grater L.; Bertens K.; Sagar L. K.; Anwar H.; Choi M. J.; Zhang Y.; Hasham M.; De Arquer F. P. G.; Hoogland S.; Wilson M. W. B.; Sargent E. H. Sequential Co Passivation in InAs Colloidal Quantum Dot Solids Enables Efficient Near Infrared Photodetectors. Adv. Mater. 2023, 35 (28), 1–8. 10.1002/adma.202301842. 37170473

[ref50] Kirkwood N.; Monchen J. O. V.; Crisp R. W.; Grimaldi G.; Bergstein H. A. C.; Du Fossé I.; Van Der Stam W.; Infante I.; Houtepen A. J. Finding and Fixing Traps in II-VI and III-V Colloidal Quantum Dots: The Importance of Z-Type Ligand Passivation. J. Am. Chem. Soc. 2018, 140 (46), 15712–15723. 10.1021/jacs.8b07783. 30375226 PMC6257620

[ref51] Hargittai M. Molecular Structure of Metal Halides. Chem. Rev. 2000, 100 (6), 2233–2302. 10.1021/cr970115u. 11749287

[ref52] Addison C. C. Inorganic Chemistry of the Main-Group Elements, Vol. 3; Royal Society of Chemistry, 1977.

[ref53] Cao X.; Zhi L.; Li Y.; Fang F.; Cui X.; Yao Y.; Ci L.; Ding K.; Wei J. Elucidating the Key Role of a Lewis Base Solvent in the Formation of Perovskite Films Fabricated from the Lewis Adduct Approach. ACS Appl. Mater. Interfaces 2017, 9 (38), 32868–32875. 10.1021/acsami.7b07216. 28853278

[ref54] Oliveri I. P.; Maccarrone G.; Di Bella S. A Lewis Basicity Scale in Dichloromethane for Amines and Common Nonprotogenic Solvents Using a Zinc(II) Schiff-Base Complex as Reference Lewis Acid. J. Org. Chem. 2011, 76 (21), 8879–8884. 10.1021/jo2016218. 21955183

[ref55] Jensen W. B. The Lewis Acid-Base Definitions: A Status Report. Chem. Rev. 1978, 78 (7), 1–22. 10.1021/cr60311a002.

[ref56] Dong A.; Ye X.; Chen J.; Kang Y. K.; Thomas G.; James M.; Kikkawa; Murray C. B. A Generalized Ligand-Exchange Strategy Enabling Sequential Surface Functionalization of Colloidal Nanocrystals. J. Am. Chem. Soc. 2011, 133 (4), 998–1006. 10.1021/ja108948z. 21175183

[ref57] Mnoyan A. N.; Kirakosyan A. G.; Kim H.; Jang H. S.; Jeon D. Y. Electrostatic Stabilized InP Colloidal Quantum Dots with High Photoluminescence Efficiency. Langmuir 2015, 31 (25), 7117–7121. 10.1021/acs.langmuir.5b00847. 26043065

[ref58] Cotton F. A.; Wilkinson G.; Murillo C. A.; Bochmann M. Advanced Inorganic Chemistry; John Wiley & Sons, 1999.

[ref59] Green M. L. H.; Parkin G. Application of the Covalent Bond Classification Method for the Teaching of Inorganic Chemistry. J. Chem. Educ. 2014, 91 (6), 807–816. 10.1021/ed400504f.

[ref60] Li Y.; Hou X.; Dai X.; Yao Z.; Lv L.; Jin Y.; Peng X. Stoichiometry-Controlled InP-Based Quantum Dots: Synthesis, Photoluminescence, and Electroluminescence. J. Am. Chem. Soc. 2019, 141 (16), 6448–6452. 10.1021/jacs.8b12908. 30964282

[ref61] Tessier M. D.; Dupont D.; De Nolf K.; De Roo J.; Hens Z. Economic and Size-Tunable Synthesis of InP/ZnE (E = S, Se) Colloidal Quantum Dots. Chem. Mater. 2015, 27 (13), 4893–4898. 10.1021/acs.chemmater.5b02138.

[ref62] Xiao P.; Zhang Z.; Ge J.; Deng Y.; Chen X.; Zhang J.-R.; Deng Z.; Kambe Y.; Talapin D. V.; Wang Y. Surface Passivation of Intensely Luminescent All-Inorganic Nanocrystals and Their Direct Optical Patterning. Nat. Commun. 2023, 14 (1), 1–11. 10.1038/s41467-022-35702-7. 36599825 PMC9813348

[ref63] Salyulev A. B.; Zakiryanova I. D. Raman Spectra of Solid, Molten, and Gaseous Gallium Trichloride. Russ. Metall. 2010, 2010 (2), 108–111. 10.1134/S0036029510020060.

[ref64] Beattie I. R.; Gilson T.; Cocking P. The Vibrational Spectrum of Ga_2_Cl_6_. J. Chem. Soc. A 1967, 702–704. 10.1039/j19670000702.

[ref65] Shamir J.; Rafaeloff R. Raman Spectra of Solid Complexes of Trihalides of Antimony and Bismuth with Trihalides of Aluminium and Gallium. J. Raman Spectrosc. 1986, 17 (6), 459–462. 10.1002/jrs.1250170606.

[ref66] Zhu G.; Angell M.; Pan C.-J.; Lin M.-C.; Chen H.; Huang C.-J.; Lin J.; Achazi A. J.; Kaghazchi P.; Hwang B.-J.; Dai H. Rechargeable Aluminum Batteries: Effects of Cations in Ionic Liquid Electrolytes. RSC Adv. 2019, 9 (20), 11322–11330. 10.1039/C9RA00765B. 35520252 PMC9062991

[ref67] Dümbgen K. C.; Leemans J.; De Roo V.; Minjauw M.; Detavernier C.; Hens Z. Surface Chemistry of InP Quantum Dots, Amine-Halide Co-Passivation, and Binding of Z-Type Ligands. Chem. Mater. 2023, 35 (3), 1037–1046. 10.1021/acs.chemmater.2c02960.

[ref68] Wehrli F. W; Wehrli S. Solution Complexes of the Aluminum Halides in Acetonitrile and Acetonitrile- Water Studied by High-Field ^27^A1 NMR. J. Magn. Reson. 1981, 44, 197–207. 10.1016/0022-2364(81)90202-X.

[ref69] Akitt J. W. Multinuclear studies of Al compounds. Prog. Nucl. Magn. Reson. Spectrosc. 1989, 21, 1–149. 10.1016/0079-6565(89)80001-9.

[ref70] Sloan J. B.; Cannon S. A.; Delionback E. C.; Dechter J. J. ^27^A1 NMR as a Test of Models of Ionic Solvation. Inorg. Chem. 1985, 24 (6), 883–886. 10.1021/ic00200a017.

[ref71] Haraguchi H.; Fujiwara S. Aluminum Complexes in Solution as Studied by Aluminum-27 Nuclear Magnetic Resonance. J. Phys. Chem. 1969, 73 (10), 3467–3473. 10.1021/j100844a056.

[ref72] Mason J. Multinuclear NMR; Springer Science & Business Media, 1987.

[ref73] Wasylishen R. E.; Wimperis S. NMR of Quadrupolar Nuclei in Solid Materials; John Wiley & Sons, 2012.

[ref74] Wen X.; Zhang J.; Luo H.; Shi J.; Tsay C.; Jiang H.; Lin Y.-H.; Schroeder M. A.; Xu K.; Guo J. Synthesis and Electrochemical Properties of Aluminum Hexafluorophosphate. J. Phys. Chem. Lett. 2021, 12 (25), 5903–5908. 10.1021/acs.jpclett.1c01236. 34152154

[ref75] Tessier M. D.; Baquero E. A.; Dupont D.; Grigel V.; Bladt E.; Bals S.; Coppel Y.; Hens Z.; Nayral C.; Delpech F. Interfacial Oxidation and Photoluminescence of InP-Based Core/Shell Quantum Dots. Chem. Mater. 2018, 30 (19), 6877–6883. 10.1021/acs.chemmater.8b03117.

[ref76] Fyfe C. A.; Bretherton J. L.; Lam L. Y. Solid-State NMR Detection, Characterization, and Quantification of the Multiple Aluminum Environments in US-Y Catalysts by ^27^Al MAS and MQMAS Experiments at Very High Field. J. Am. Chem. Soc. 2001, 123 (22), 5285–5291. 10.1021/ja003210k. 11457391

[ref77] Rosales-Sosa G. A.; Masuno A.; Higo Y.; Inoue H.; Yanaba Y.; Mizoguchi T.; Umada T.; Okamura K.; Kato K.; Watanabe Y. High Elastic Moduli of a 54Al_2_O_3_-46Ta_2_O_5_ Glass Fabricated via Containerless Processing. Sci. Rep. 2015, 5 (1), 1–8. 10.1038/srep15233. PMC4606732 26468639

[ref78] Tu B.; Liu X.; Wang H.; Wang W.; Zhai P.; Fu Z. Combining ^27^Al Solid-State NMR and First-Principles Simulations To Explore Crystal Structure in Disordered Aluminum Oxynitride. Inorg. Chem. 2016, 55 (24), 12930–12937. 10.1021/acs.inorgchem.6b02360. 27989198

[ref79] Soubayrol P.; Dana G.; Man P. P. Aluminium-27 Solid-state NMR Study of Aluminium Coordination Complexes of Alizarin. Magn. Reson. Chem. 1996, 34, 638–645. 10.1002/(SICI)1097-458X(199608)34:8<638::AID-OMR926>3.0.CO;2-5.

[ref80] Bleam W. F.; Dec S. F.; Frye J. S. ^27^A1 Solid-state Nuclear Magnetic Resonance Study of Five-Coordinate Aluminum in Augelite and Senegalite. Phys. Chem. Miner. 1989, 16 (8), 817–820. 10.1007/BF00209706.

