# Supplementary material for: Metal–Solvent Complex Formation at the Surface of InP Colloidal Quantum Dots
Source: J Am Chem Soc. 2024 Apr 26;146(18):12808–18. doi: 10.1021/jacs.4c03325 (PMC11082887; doi:10.1021/jacs.4c03325)
Supplement: Supplementary file 1 — ja4c03325_si_001.pdf [file ja4c03325_si_001.pdf]

Metal-Solvent Complex Formation at the Surface of InP Colloidal Quantum Dots

Supporting Information

Yun Hai,[1] Kushagra Gahlot,[1] Mark Tanchev,[2] Suhas Mutalik,[1] Eelco K. Tekelenburg,[1] Jennifer Hong,[1] Majid Ahmadi,[1] Laura Piveteau,[2] Maria Antonietta Loi,[1] Loredana Protesescu[1,*]

1-Zernike Institute for Advanced Materials, University of Groningen, Nijenborgh 4, Groningen, 9747AG, The Netherlands.

2-Institute of Chemistry and Chemical Engineering, École Polytechnique Fédérale de Lausanne, 1015 Lausanne, Switzerland.

E-mail: l.protesescu@rug.nl

Table of Contents

I. Experimental Sections 2

II. Characterization 3

III. Theoretical Calculation of InP QDs 5

IV. Supporting tables and figures 7

Table S1. The theoretical calculation table from 2 different batch samples. 7

Table S2. The parameters of ligand exchange with metal halides. 8

Table S3. The original data of ICP-MS of InP QDs after LE with MI_3. 9

Figure S1. Photographs of ligand-exchange phase transfer. 10

Figure S2. X-ray diffraction patterns. 11

Figure S3. HAADF-STEM image and the size distribution of InP/MAc QDs after LE with AlI_3. 12

Figure S4. The first excitonic peak shift of InP/MAc and InP/OAm QDs after LE with MX_3. 13

Figure S5. ICP-MS results 14

Figure S6. PL spectra of InP/MAc QDs after LE with MX_3 and InP/OAm QDs after LE with MX_3. 15

Figure S7. Screening experiments of different ratios of metal halides to organic ligands 16

Figure S8. The decay time of InP/MAc and InP/OAm QDs after LE with MI_3. 17

Figure S9. Raman spectra of InP-MI_3 QDs from InP/MAc QDs and InP/OAm QDs. 18

Figure S10. FTIR spectra of InP/MAc and InP/OAm QDs after LE with MI_3. 19

Figure S11. FTIR spectra of InP/MAc and InP/OAm QDs LE with of $InCl_3$. 20

Figure S12. Liquid-state ^{27}Al NMR of AlI_3 salt in different Lewis base solvents. 21

Figure S13. Liquid-state ^{27}Al NMR of InP/MAc and InP/OAm QDs after LE with AlI_3 in MFA. 22

Figure S14. Liquid-state ^{27}Al NMR of AlI_3 in MFA and the AlI_3 in MFA/ H_2O solution. 23

Figure S15. Zeta potential value of InP/MAc and InP/OAm QDs after LE with MI_3. 24

Figure S16. Zeta potential spectra of InP QDs after LE with $Al(NO_3)_3$ and $Al(ClO_4)_3$. 25

Figure S17. Colloidal dispersity of InP/$[In(MFA)_6]^{3+}$ QDs in different polar solvents. 26

Figure S18. The absorbance spectra of InP/$[In(MFA)_6]^{3+}$ QDs inks in MFA. 27

References 28

I. Experimental Sections

Chemicals

Indium (III) acetate (99.99%, Sigma), indium (III) chloride (99.999%, anhydrous, Alfa Aesar), tris(trimethylsilyl)phosphine ($P(SiMe_3)_3$, 98%, Acros), tris(diethylamino)phosphine ($P(NEt_2)_3$), 97%, Sigma), trioctylphosphine (TOP, 97%, Sigma), myristic acid (MAc, 99%, Sigma), 1-octadecene (ODE, 90%, Sigma), oleyl amine (OAm, 80-90%, Acros), indium (III) bromide (99.99%, anhydrous, Acros), indium (III) iodide (99.998%, anhydrous, Acros), gallium (III) chloride (99.99+%, anhydrous, Acros), gallium (III) bromide (99.99%, anhydrous, Sigma), gallium (III) iodide (99.999%, ultra-dry, Alfa Aesar), aluminum (III) chloride (99.99%, Sigma), aluminum (III) bromide (98+%, extra pure, anhydrous, Acros), aluminum (III) iodide (99.99%, Acros), aluminum (III) nitrate hydrate (99.8%, Sigma), gallium(III) nitrate hydrate (99.9998% trace metal basis, Acros), indium(III) nitrate hydrate (99.999% trace metals basis, Sigma), aluminum (III) perchlorate nonahydrate (Reagent grade, Alfa Aesar), gallium (III) perchlorate hydrate (99.999% trace metals basis, Sigma), indium(III) perchlorate octahydrate (99.9%, Strem), toluene (AR, Macron Fince Chemicals), n-methylformamide (MFA, 99%, Sigma), hexane (AR, Macron Fince Chemicals), acetone (AR, Macron Fince Chemicals), n-methyl acetamide (MAA, +99%, Sigma), formamide (FA, 99%, Sigma), propylene carbonate (PC, +98.0%, TCI), dimethyl sulfoxide (DMSO, anhydrous +99.9%, Sigma), dimethyl formamide (DMF, anhydrous, Sigma), n-ethylformamide (EFA, 99.0%GC, Sigma), acetonitrile (ACN, anhydrous, Sigma), methanol (MeOH, anhydrous 99%, Sigma), hexamethylphosphoramide (HMPA, anhydrous, Sigma), ethanol (EtOH, absoulte, Avantor), isopropanol (i-PrOH, ACS reagent, Sigma), n-butanol (n-BuOH, anhydrous 99.9%, Sigma), DMSO-D_6 (99%, Alfa Aesar), toluene-D_8 (99.6 atom% D, Sigma), deuterium oxide (D_2O, 99.9 atom% D, Sigma). ODE and OAm were dried under vacuum at 120 ^oC for 1h and stored in the glove box.

Synthesis of InP QDs capped with X- and L-type organic ligands

InP QDs capped with X-type ligand (myristic acid, MAc) were synthesized by modifying the method of Peng et al.[1] Indium (III) acetate (0.876 g, 3.0 mmol), myristic acid (2.055 g, 9.0 mmol), and ODE (100 mL) were mixed in a 250 mL three-neck flask. The mixture was degassed under the vacuum at 120 ^oC for 1 h and heated to 150 ^oC under nitrogen flow. Then, TOP (15 mL) and $P(SiMe_3)_3$ (444.3 μL, 1.5 mmol) in 5mL ODE were injected into the flask very fast and successively. The reaction temperature was increased to 270 ^oC and kept at this temperature for 40 min. After that, the reaction was cooled down to room temperature by removing the heating mantle. The crude products were washed and purified 3 times using toluene and ethanol, and the final InP QDs product was stored in hexane for the ligand exchange step. The reaction equation is shown below:

$$In(Ac)_3 + 3HMy + P(SiMe_3)_3 \xrightarrow[270^oC]{TOP/ODE} InP + 3MySiMe_3 + 3HAc\uparrow$$

$$Ac: CH_3COO;\ My: CH_3(CH_2)_{12}COO;\ Me: CH_3$$

Equation-1

InP QDs capped with L-type ligand (oleylamine, OAm) were synthesized according to the method of Zeger Hens et al.[2] Indium (III) chloride (2.017 g, 9.12 mmol) and OAm (100 mL) were mixed in a 250 mL three-neck flask. The mixture was dissolved under the vacuum at 120 °C for 1-2 h until getting a clear solution. The reaction temperature was raised to 180 °C under nitrogen flow, and $(DEA)_3P$ (9.27 mL, 32.83 mmol) was quickly injected and kept at this temperature for 20 min. Then, the reaction was cooled down to room temperature by removing the heating mantle. The crude products were washed and purified 3 times using toluene and ethanol, and the final InP QDs product was stored in hexane for the ligand exchange step.

$$InCl_3 + 4P(NEt_2)_3 + 12R\text{-}NH_2 \xrightarrow[180^oC]{} InP + 3P(NHR)_4\cdot Cl + 12Et_2NH\uparrow$$

$$Et: CH_3CH_2;\ R: C_{18}H_{35}$$

Equation-2

Ligand exchange with metal salts

Typically, 24.3 μmol metal salts were dissolved in 6 mL MFA getting a clear solution. The metal halide solution was mixed with a 6 mL hexane solution of InP QDs capped with organic ligands (6 mg). The biphasic system was stirred vigorously for 30 minutes to 24 h until the complete migration of InP QDs from the hexane phase (nonpolar) to the MFA phase (polar). Then, the nonpolar phase was discarded and the polar phase was purified 4 times with hexane (centrifugation, 5000 rpm @ 3 min). To remove the free metal salts, InP QDs capped with metal halide ligands were precipitated from the MFA phase by adding an anti-solvent (acetone+toluene), and then redispersed in MFA.

II. Characterization

Absorption spectra were collected from a UV-Vis-NIR spectrophotometer (Shimadzu UV-3600). InP QDs capped with organic ligands were dispersed in hexane and InP QDs capped with metal halides were dispersed in MFA. All samples were measured in quartz cuvettes.

Steady-state photoluminescence measurements were performed on a Horiba Scientific Jobin Yvon 8 spectrometer equipped with a PMT detector. The excitation wavelength is 480nm, provided by a 450W Xenon lamp dispersed with a monochromator.

Time-resolved photoluminescence (TRPL): the samples were excited at 3.1 eV (400 nm) using the second harmonic of a mode-locked Ti: sapphire laser (Mira 900, coherent). The repetition rate of 76 MHz of the laser was reduced using an optical pulse selector. The excitation beam was spatially limited by an iris to 2 mm and was focused with a lens (150 mm focal length). The fluence was adjusted using neutral density filters and spectra were taken in reflection geometry. The photoluminescence was collected using an achromatic doublet, where 425 nm and 435 nm long pass filters were used to block

the scattered laser light. Steady-state spectra were recorded with a Hamamatsu EM-CCD camera that was spectrally calibrated. The spectrograph was equipped with a 50 l/mm grating. A Hamamatsu streak camera working in single sweep mode was used for the time-resolved traces.

X-ray diffraction (XRD) powder XRD patterns were measured on a Bruker D8 Advanced diffractometer with Cu Kα radiation (λ = 1.54 Å) and a Lynxeye detector. All samples were measured in powder after being dried in a vacuum oven.

Scanning transmission electron microscopy (STEM) was performed using an aberration-corrected Thermo Fisher Scientific Themis Z STEM operating at 300 kV. The TEM samples were prepared on an ultrathin grid with 400 mesh, Cu (Ted Pella, Inc. 01822-F) which are wrapped with graphene on one side. The Sample was then drop-casted on the graphene side of the grid which is then sandwiched between two graphene layers using the other grid. The TEM grid was dried overnight in the antechamber of the glove box.

Fourier Transform Infrared (FTIR) spectroscopy was performed on a Shimadzu IR Spirit FTIR spectrometer in the range of 500-4000 cm^{-1} with a resolution of 1.43 cm^{-1}. All samples were measured in powder after being dried in a vacuum oven.

Liquid-state nuclear magnetic resonance (NMR) spectra were measured with an Agilent 400 MHz NMR. ^{27}Al NMR was recorded with 500 scans (-250ppm to 250ppm) and a relaxation time of 2s, the solvent is DMSO-d_6, and the NMR tube is a 5mm Quartz tube from NORELL.

Solid-state NMR ^{31}P solid-state NMR spectra were recorded on a 500 MHz Bruker spectrometer (11.7 T) equipped with an Avance III HD console and a 3.2 mm triple channel broad-band CPMAS probe. ^{27}Al solid-state NMR spectra were recorded on a 900 MHz Bruker spectrometer (21.1 T) equipped with an Avance NEO console and a 3.2 mm triple channel HCN CPMAS probe. Samples were packed into 3.2 mm zirconia rotors and spun at 20 and 24 kHz spinning speed using dry nitrogen gas at room temperature for ^{31}P and ^{27}Al NMR measurements respectively. ^{31}P solid-state NMR spectra were recorded with one-pulse excitation using 90° = 2.75 μs pulses and heteronuclear proton decoupling during acquisition. T_1 relaxation times were determined from saturation recovery experiments und recovery delays were set to 5*T_1 for every sample to guarantee quantitative excitation. ^{31}P chemical shift were referenced relative to 85% H_3PO_4 using the secondary reference $NH_4H_2PO_4$ at 1.33 ppm. ^{27}Al solid-state NMR spectra were recorded using a single, short hard pulse of 0.4 μs equivalent to a flip angle inferior to 7.5°. This allowed a quantitative, homogeneous excitation of the whole signal, including the satellite transitions. Proton decoupling had no effect on signal line shapes and was therefore omitted to preserve the probe. Up to 2048 transients were cumulated per spectrum, using recycle delays equivalent to T_1. Flip angles in absence of quadrupole interaction were obtained from 1.1 *m* $Al(NO_3)_3$ in H_2O.

Raman spectra were collected via a confocal Raman microscope**.** All InP QDs samples were measured in powder form on the silicon wafer. A single scan measurement is generated by the WiRE spectral acquisition wizard. The laser was used at a wavelength of 785 nm (power 5%), the grating is 1800 l/mm

(vis), the detector is Renishaw Centrus 1UTR61, the scan type is continuous scan, exposure time is 10.0 s, accumulations are 5 times, the focus mode is regular, and the spectral range is 50-800 cm^{-1}.

Zeta Potential measurements were performed using Zetasizer Nano ZS (Malvern Instruments, Inc.). Colloidal solutions were measured using a dip cell setup with Pd electrodes and a quartz cuvette. InP QDs capped with organic ligands dispersed in hexane and the concentration is around 2.0 mg/mL; InP QDs capped with metal halides were dispersed in MFA and the concentration is 0.5-1.0 mg/mL. Each sample was measured three times. Typical measurements of zeta potential included several scans of 100 runs each in the high-resolution mode.

ICP-MS (inductively coupled plasma mass spectrometry) data collected by the Thermo iCAP TQ. The measuring and analysis details are that around 10 mg of each sample were added to 100 µL 32.5% HNO_3. Slightly heated it (in hot water) and used ultrasonics to get the best transfer from the material to the HNO_3. Then 400 µL ultrapure water was mixed and centrifuged. Then, 200 µL of standard or Blanc (ultrapure water) or 20 µL of diluted sample (1000x diluted) was pipetted in a mixture solution. So, the sample has a dilution of 10000x. added 3.5 mL of dilution solvent (0.1% HNO_3 in ultrapure water and 25 µL/L Be, Sc, Ge, Y, Ir (1000 mg/L)) was added and measured.

III. Theoretical Calculation of InP QDs

Size of InP QDs: the size of InP QDs (diameter) is calculated by the empirical formula:[3]

$$\boldsymbol{Eg} = 1.35 + \frac{1}{0.119 \times d^2}$$

Where E_g is the bandgap of InP QDs determined by the first excitonic absorption peak.

The molar absorption coefficient of InP QDs: the molar absorption coefficient is calculated by the correlation formula:[4, 5]

$$\boldsymbol{\varepsilon = 3046.1\times d^3 - 76532\times d^2 + 5.5137\times 10^5\times d - 8.9839\times 10^5}$$

Where d is the diameter of InP QDs.

Molar Concentration of InP QDs: the concentration of InP QDs is calculated from Beer-Lambert Equation:[5]

$$\boldsymbol{A = \varepsilon\cdot b\cdot C}$$

Where A is the absorbance, ε is the molar absorption coefficient ($L\cdot mol^{-1}\cdot cm^{-1}$), b is the length of pathlength (cm), and $C_{cuvette}$ is the concentration of InP QDs solution in the cuvette (mol/L).

After getting the $C_{cuvette}$, calculate the concentration of InP QDs in stock solution C_{stock}:

$$\mathbf{C_{\mathit{stock\ solution}} = diluted\ factor \cdot C_{\mathit{cuvette}}}$$

Mass concentration of InP QDs: Before calculating the mass concentration of InP QDs, we need to know the molar weight of a single InP QD. We assume that every single InP QD is a sphere. The density (ρ) of InP is 4.81 g/cm^3, and the Avogadro constant (N_A) is 6.02214076×10^{23} mol^{-1}, so the $Mw_{(single\ InP\ QD)}$ is calculated by the following:

$$\mathbf{Mw_{\mathit{(single\ InP\ QD)}} = 4/3\cdot \pi\cdot(d/2)^3\cdot\rho\cdot N_A\cdot 10^{-21}}$$

$$\mathbf{Mass\ concentration = C_{\mathit{stock}\ solution} \cdot Mw_{\mathit{(single\ InP\ QD)}}}$$

Estimating the number of In atoms on the surface and ligand amounts: using the method outlined by Peterson et al.[6] we calculated the maximum number of indium myristates that could pack on the surface of our quantum dots based on indium sites based on the packing efficiency of a circle inside a square. The adapted geometric equation is[5]

$$N = \frac{\pi}{4} \cdot \frac{4\pi R_{QD}^{2}}{\pi R_{P3-}^{2}}$$

where the R_{QD} is the radius of InP QDs, and R_{P3-}^{2} is the radius of a phosphorus anion has been reported as 0.212 nm.[5] This N number can be used as an approximation of the number of In atoms on the surface of InP QDs. (N_P=N_{In})

Thus, the ligand amount of myristate per QD can be calculated as: $\mathbf{N_{L,\,QD} = N_{In}}$

Here we consider that one In atom on the surface is coordinated by one myristate ligand through the bidentate chelating. And the same coordination way, one In aton on the surface is coordinated by one oleylamine ligand through monodentate chelating.[7]

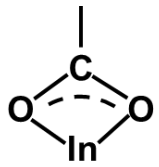

Chelating bidentate

Ligand coverage/nm^2 and /QD: the ligand coverage per InP QD and ligand/nm^2 are calculated by:

Ligand coverage/*nm*^{2}= $N_{L,\,QD}$/4 · π· (d/2)^2

Ligand coverage/*QD*= $N_{L,\,QD}$ / QD

The amounts of ligands in a given solution: in a given solution (*V*), the total amount of ligands (mol) can be calculated below: $\boldsymbol{n_{total} = C_{stock\ solution} \cdot N_{L,\,QD} \cdot V}$

where $\boldsymbol{C_{stock\ solution}}$ is the molar concentration of InP QDs, and $\boldsymbol{N_{L,\,QD}}$ is the ligands number per QD.

IV. Supporting tables and figures

Table S1. The theoretical calculation table from 2 different batch samples.

**Parameters**	**Sample 1 (InP/MAc)**	**Sample 2 (InP/OAm)**
First excitonic peak (nm)	520	540
E_g (eV)	2.38	2.29
Size (D, nm)	2.85	2.98
Molar absorption coefficient (ε, L·mol-1cm-1)	121967.71	145746.12
Absorbance (A)	0.346159	0.611277
Molar concentration in cuvette (mol/L)	2.83×10^-6	4.19×10^-6
Diluted factor	66.666	66.666
Molar concentration in stock solution (mol/L)	0.000189208	0.000279608
Molar weight of single InP QD (g/mol)	35123.4952	40154.62387
Mass Concentration in stock solution (mg/mL)	6.65	11.23
Number of In atoms on the surface per InP QD	142.0	155.2
Number of ligands per InP QD (N_L,QD)	142.0	155.2
Ligands coverage/nm^2 per InP QD	5.6	5.6
The volume of a given solution (mL)	0.903	0.535

Table S2. The parameters of ligand exchange with metal halides.

InP QDs Samples	Metal Halides	Molar ratio (MAc/OAm: MX_3)	The molar amount of MAc/OAm (μmol)	The molar amount of MX_3 (μmol)	Time (h)
Sample 1 InP/MAc	$AlCl_3$	1:1	24.3	24.3	3
	$AlBr_3$	1:1	24.3	24.3	3
	AlI_3	1:1	24.3	24.3	3
	$GaCl_3$	1:1	24.3	24.3	0.5
	$GaBr_3$	1:1	24.3	24.3	0.5
	GaI_3	1:1	24.3	24.3	0.5
	$InCl_3$	1:1	24.3	24.3	20
	$InBr_3$	1:1	24.3	24.3	20
	InI_3	1:1	24.3	24.3	20
Sample 2 InP/OAm	$AlCl_3$	1:1	23.2	23.2	3
	$AlBr_3$	1:1	23.2	23.2	3
	AlI_3	1:1	23.2	23.2	3
	$GaCl_3$	1:1	23.2	23.2	0.5
	$GaBr_3$	1:1	23.2	23.2	0.5
	GaI_3	1:1	23.2	23.2	0.5
	$InCl_3$	1:1	23.2	23.2	4
	$InBr_3$	1:1	23.2	23.2	4
	InI_3	1:1	23.2	23.2	4

Table S3. The original data of ICP-MS of InP QDs after LE with MI_3.

Num.	Samples	^{115}**In** (μmol/mg)	^{31}**P** (μmol/mg)	^{27}**Al** (μmol/mg)	^{71}**Ga** (μmol/mg)	^{127}**I** (μmol/mg)
1	InP/MAc	0.6313043	0.3451613	-	-	-
2	InP←AlI_3	2.7652174	2.1677419	1.72963	-	0.2629921
3	InP←GaI_3	2.4086957	1.8064516	-	1.676056	0.3110236
4	InP←InI_3	4	1.6612903	-	-	0.4007874
5	InP/OAm	1.1565217	0.9967742	-	-	-
6	InP←AlI_3	3.0695652	3.1225806	1.122222	-	0.292126
7	InP←GaI_3	0.3947826	0.3903226	-	0.079577	0.2181102
8	InP←InI_3	1.2086957	0.7387097	-	-	0.184252

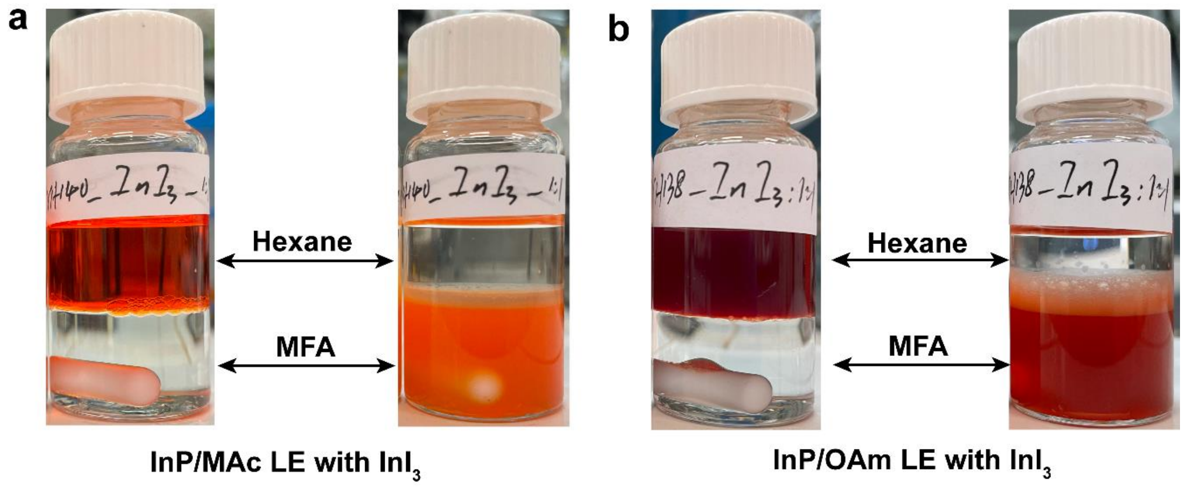

Figure S1. Photographs of ligand-exchange phase transfer (a) InP/MAc QDs LE with InI_3 salt and (b) InP/OAm QDs LE with InI_3 salt.

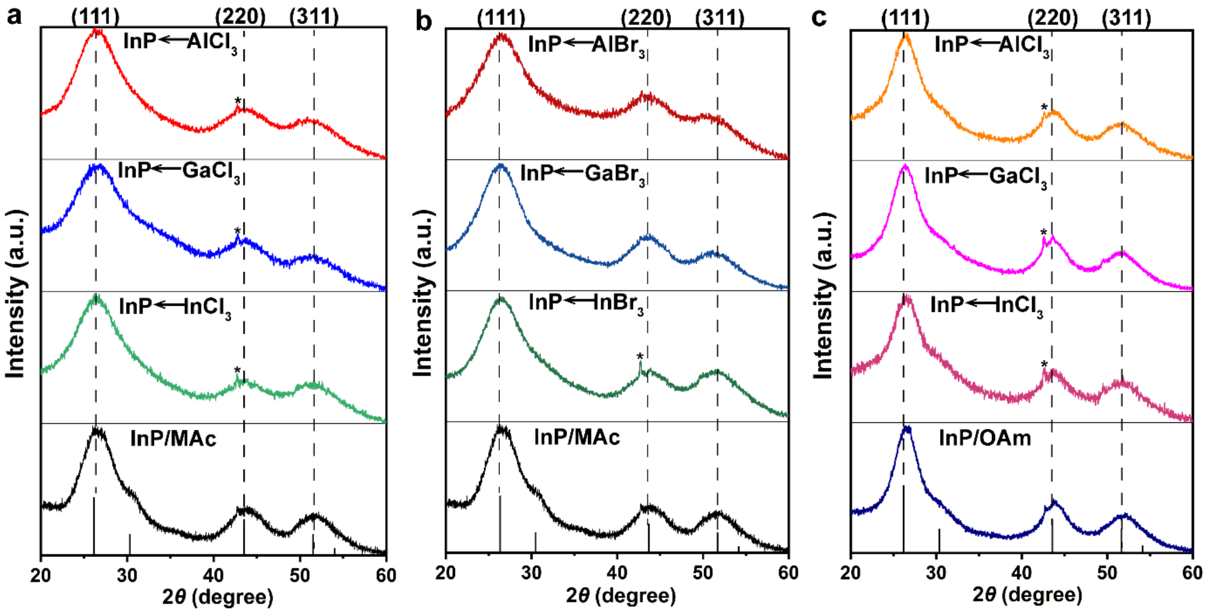

Figure S2. X-ray diffraction patterns: (a) InP/MAc QDs after LE with MCl_3 salts (b) InP/MAc QDs after LE with MBr_3 salts, and (c) InP/OAm QDs after LE with MCl_3 salts. * marked peaks are from the blade of the XRD instrument.

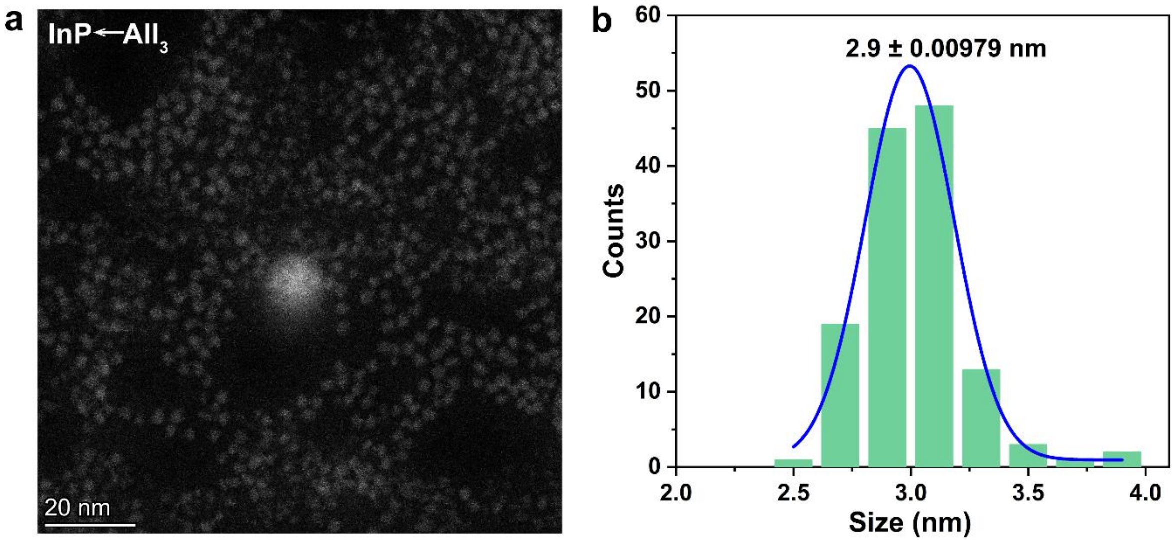

Figure S3. (a) HAADF-STEM image and (b) the size distribution of InP/MAc QDs after LE with AlI_3 salt.

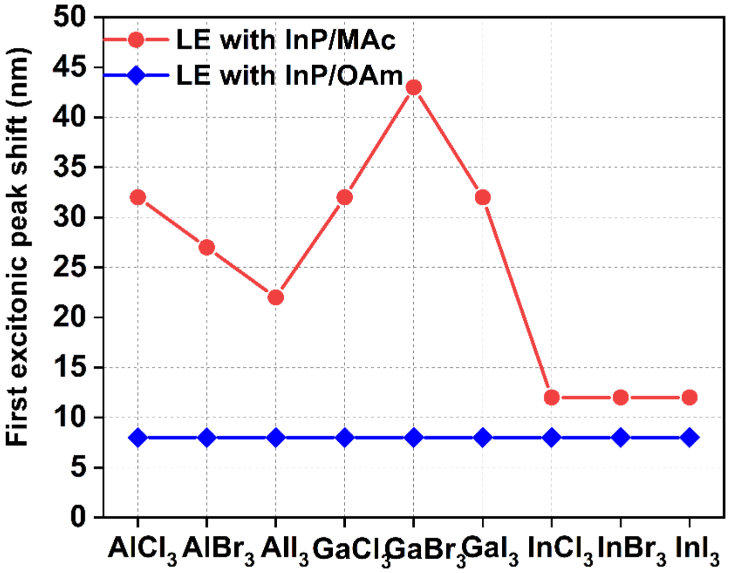

Figure S4. The first excitonic peak shift of InP/MAc and InP/OAm QDs after LE with MX_3 salts.

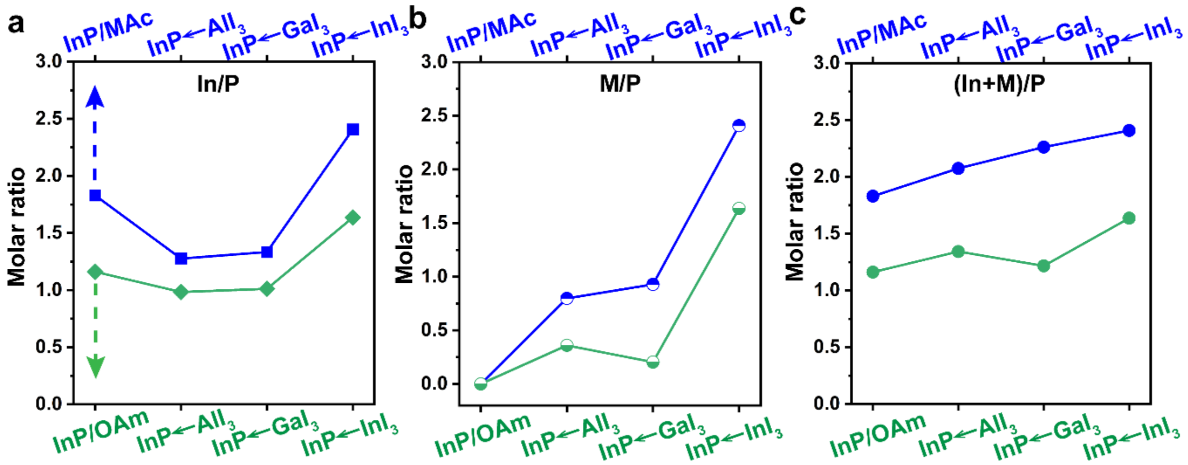

Figure S5. ICP-MS results: (a) In/P molar ratio, (b) M/P molar ratio (M=Al, Ga, In), and (c) (In+M)/P molar ratio. The M is from external metal halides. The blue curve is for InP/MAc QDs side and the green curve is for the InP/OAm QDs side.

ICP-MS measurement was employed to track the element variation of InP QDs after ligand exchange, as shown in Figure S5. We can see that the pristine InP/MAc QDs and InP/OAm QDs are indium rich and the In/P ratios are 1.82 and 1.16, respectively, which are similar to the literature. After ligand exchange, the In/P ratios in InP←AlI_3 QDs and InP←GaI_3 QDs from both sides declined compared with InP/MAc QDs and InP/OAm QDs, which can be explained that a portion of surface indium atoms stripped off with organic ligands causing a decrease of indium ratio and which is consistent with the results of the blue shift in absorbance spectra. The In/P ratios in InP←InI_3 QDs are much higher than that of InP←AlI_3 QDs and InP←GaI_3 QDs on both sides, which are attributed to the contribution of surface InI_3 ligands. Moreover, the Al/P and Ga/P ratios on the InP/MAc side are higher than the respective ratios on the InP/OAm QDs side, which can be interpreted that more Al and Ga elements penetrated the surface layer of InP/MAc than InP/OAm, and this also can be confirmed from that InP←AlI_3 QDs and InP←GaI_3 QDs from InP/MAc QDs have a larger blue shift in absorbance spectra than from InP/OAm QDs.

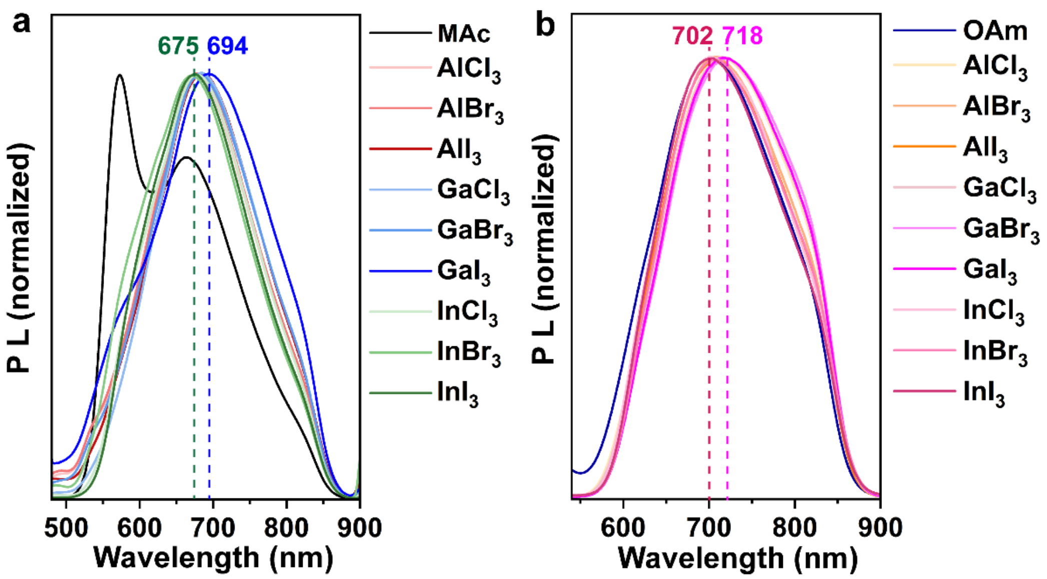

Figure S6. PL spectra of (a) InP/MAc QDs after LE with MX_3 salts and (b) InP/OAm QDs after LE with MX_3 salts.

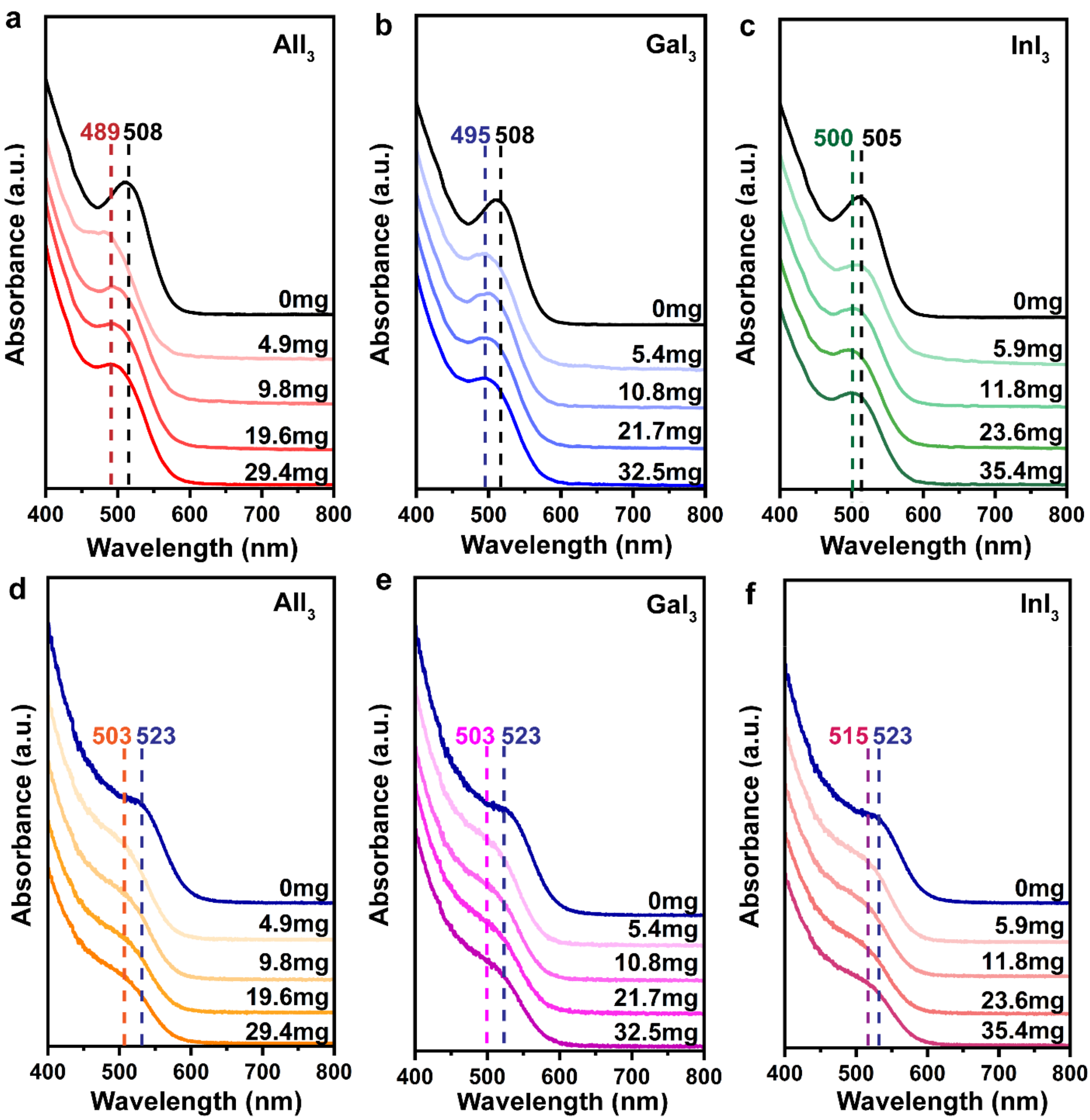

Figure S7. Screening experiments of different ratios of metal halides to organic ligands: (a-c) InP/MAc LE with MI_3 salts; (d-f) InP/OAm LE with MI_3 salts.

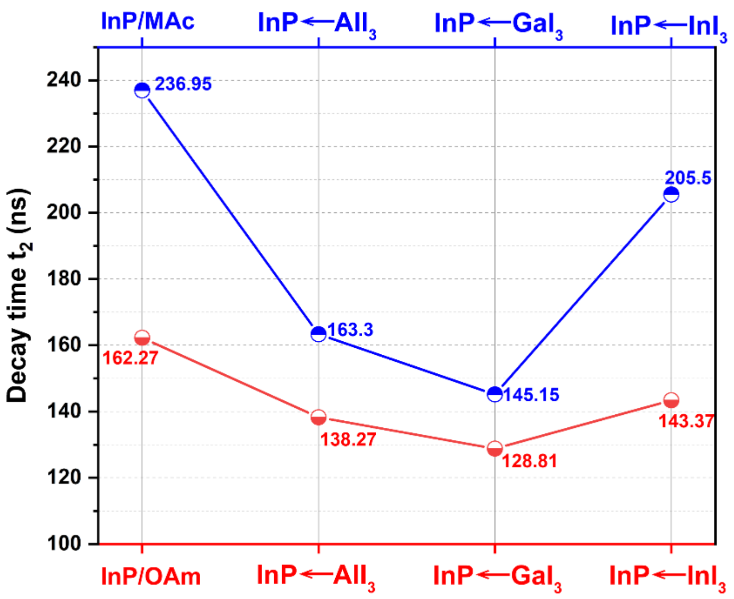

Figure S8. The decay time of InP/MAc QDs after LE with MI_3 salts (blue) and InP/OAm QDs after LE with MI_3 salts.

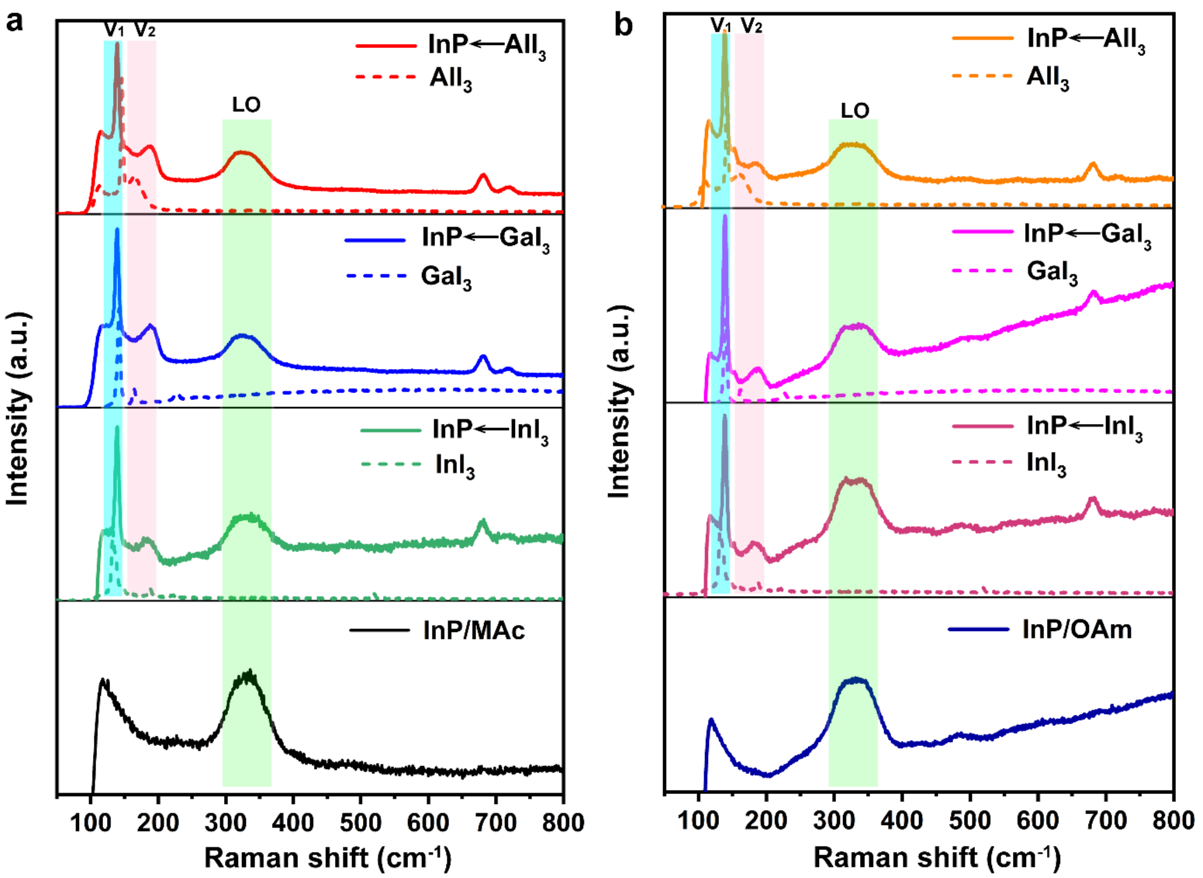

Figure S9. Raman spectra of (a) InP/MAc QDs after LE with MI_3 salts and (b) InP/OAm QDs after LE with MI_3 salts.

For the InP/MAc and InP/OAm QDs, their longitudinal optical (LO) modes are detected at around 336 cm^{-1} which is the characteristic signal of InP QDs and it is consistent with the literature.[8, 9] This LO signal appears in all InP⟵MI_3 QDs, which indicates that the original crystal structure of InP QDs preserved after the ligand exchange. Moreover, to track the characteristics of metal halides, the Raman spectra of all pure metal iodides (AlI_3, GaI_3, and InI_3) were measured and presented by a dashed line. All metal iodides show two similar vibrational signals V_1 (~140 cm^{-1}) and V_2 (~167 cm^{-1}) which are the typical Raman signals of III-group metal halides in the solid phase, and which are in correspondence with the reports.[10-13].

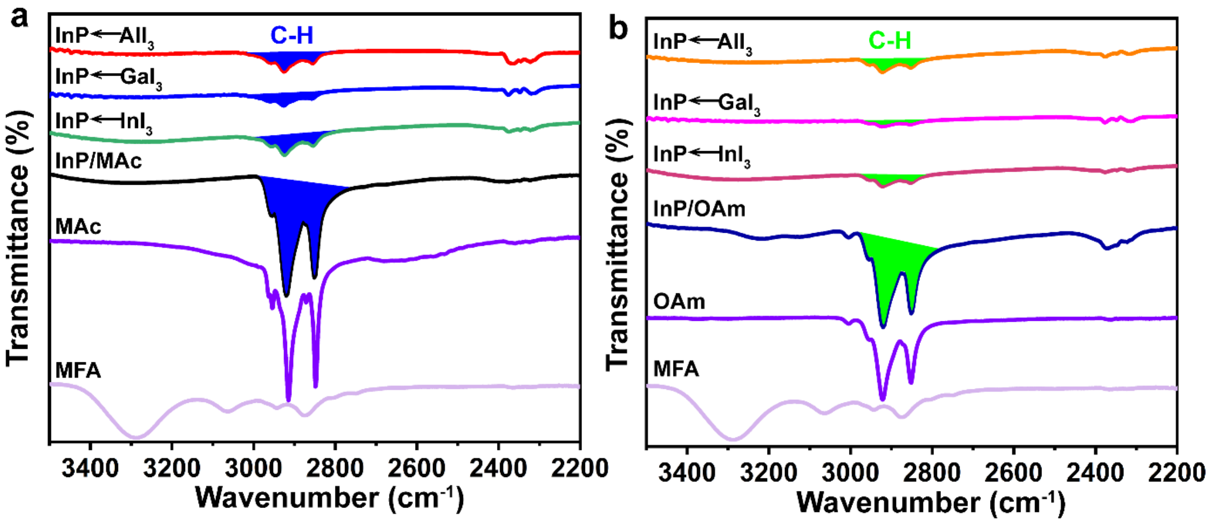

Figure S10. FTIR spectra of (a) InP/MAc QDs after LE with MI_3 salts and (b) InP/OAm QDs after LE with MI_3 salts.

FTIR spectroscopy was applied to qualitatively analyze the surface ligands of InP QDs before and after ligand exchange. As shown in Figure S10, the strong signals (2917 and 2848 cm^{-1}) appear in both InP/MAc and InP/OAm QDs spectra which can be assigned to the characteristic vibration peaks of C-H stretching of MAc and OAm, respectively. After ligand exchange with metal iodides, there are still the C-H signals remaining in all InP←MI_3 QDs, but the area formed by C-H peaks from all InP←MI_3 QDs is much smaller than that of InP/MAc QDs and InP/OAm QDs, which can be used to qualitatively demonstrate that most parts of organic ligands were removed by metal iodides. The left small portion can be ascribed to some binding or free organic ligands and the solvent MFA which interacts with the metal halide ligands and is difficult to eliminate from the system by a washing step, which coincides with the reports.[14, 15]

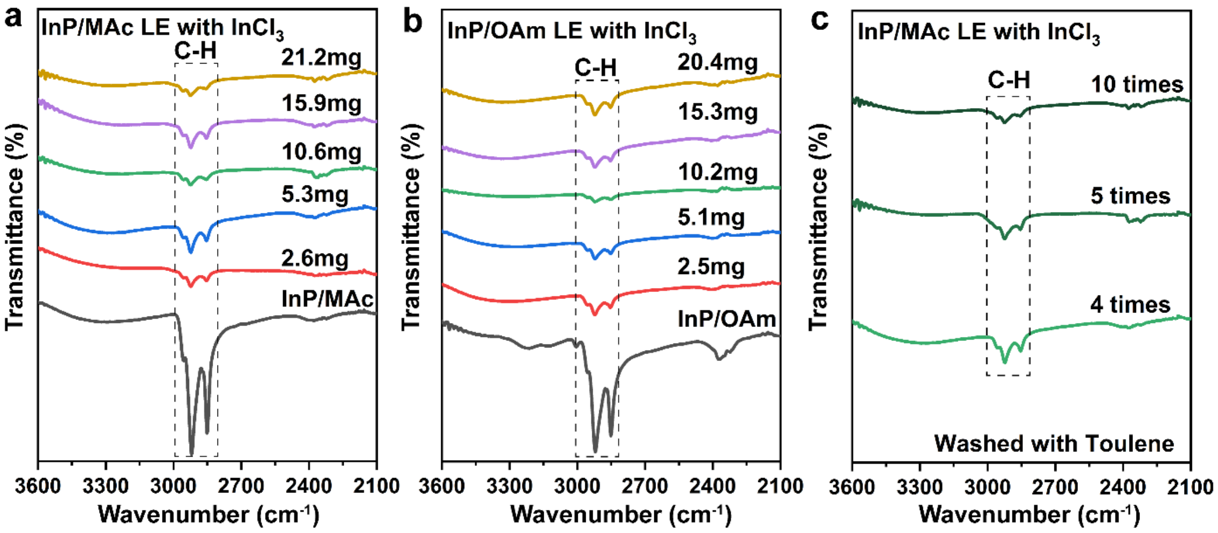

Figure S11. FTIR spectra of (a) InP/MAc LE with different initial amounts of $InCl_3$ salt, (b) InP/OAm LE with different initial amounts of $InCl_3$ salt, and (c) InP/MAc QDs after LE with $InCl_3$ salt washed at different times with toluene.

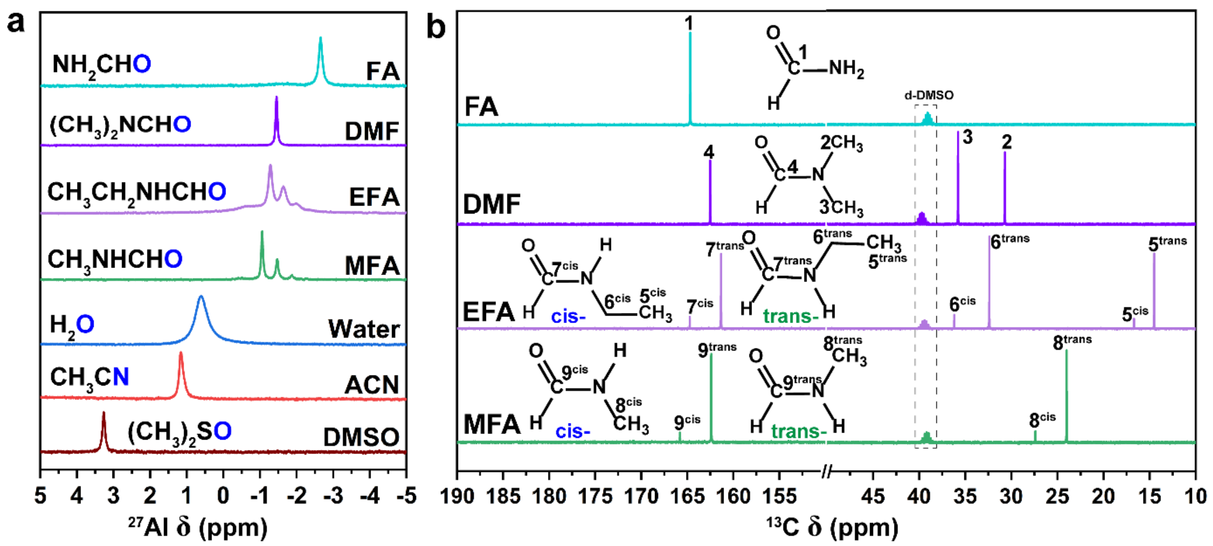

Figure S12. Liquid-state ^{27}Al NMR of AlI_3 salt in different Lewis base solvents, only $AlCl_3$ salt was chosen for EFA because AlI_3 salt cannot fully dissolve in EFA. (b) Liquid-state ^{13}C NMR of FA, DMF, EFA, and MFA.

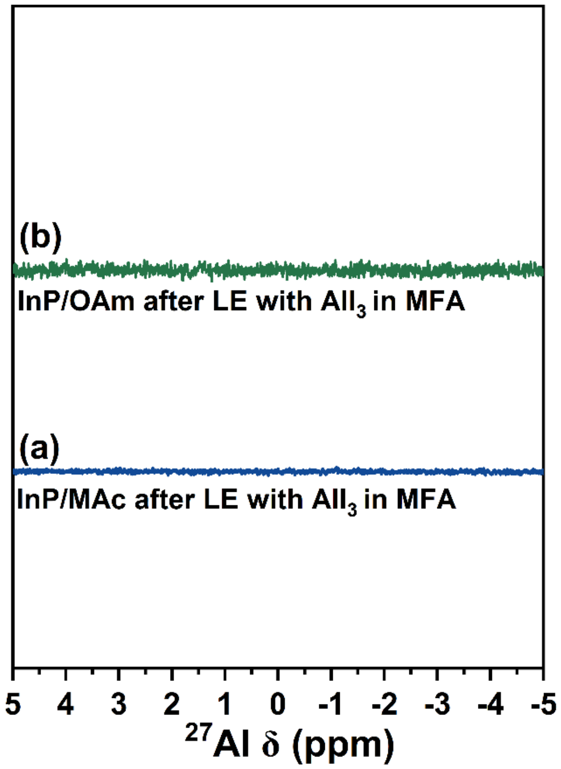

Figure S13. Liquid-state ^{27}Al NMR of (a) InP/MAc after LE with AlI_3 salt in MFA and (b) InP/OAm after LE with AlI_3 salt in MFA.

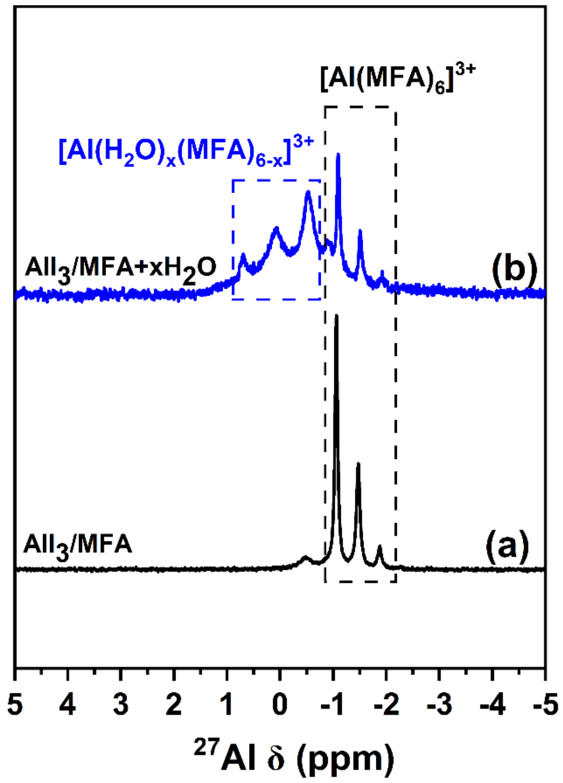

Figure S14. Liquid-state ^{27}Al NMR of AlI_3 salt in MFA (a) and adding around 20 uL H_2O to the AlI_3/MFA solution (b).

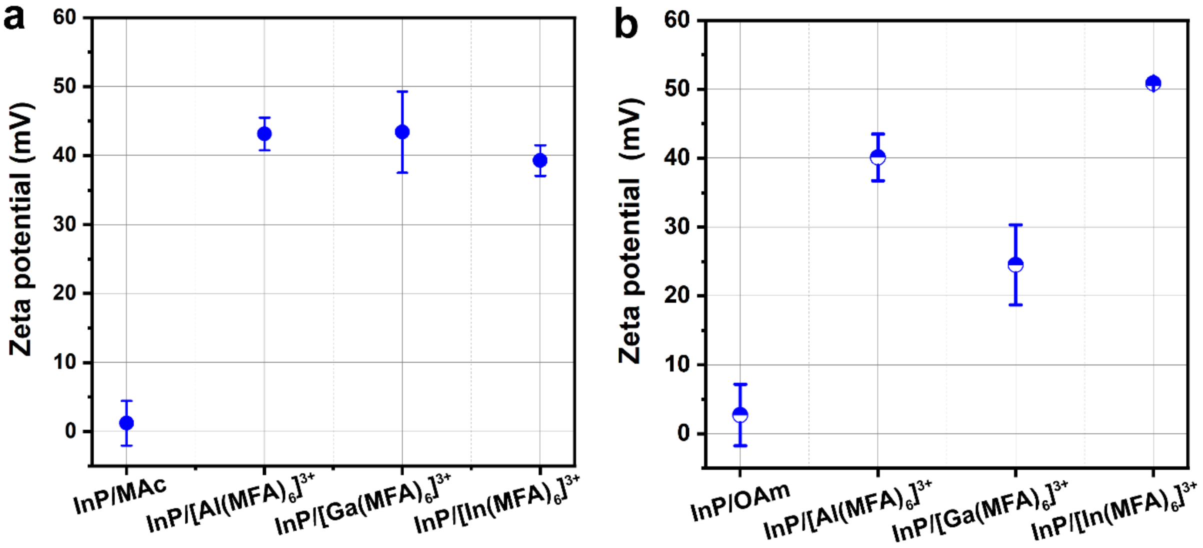

Figure S15. Zeta potential value of (a) InP/MAc QDs after LE with MI_3 salts and (b) InP/OAm QDs after LE with MI_3 salts.

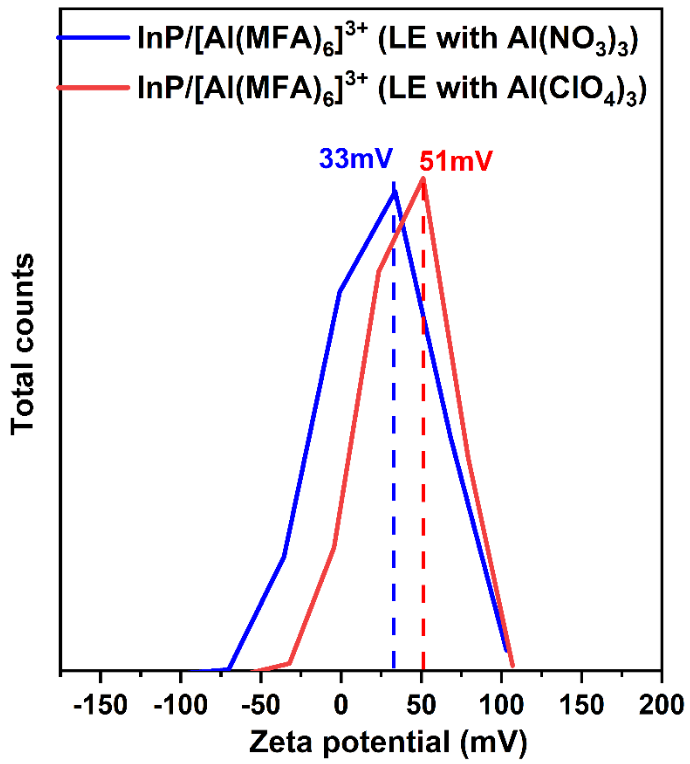

Figure S16. Zeta potential spectra of InP QDs after LE with $Al(NO_3)_3$ and $Al(ClO_4)_3$ salts.

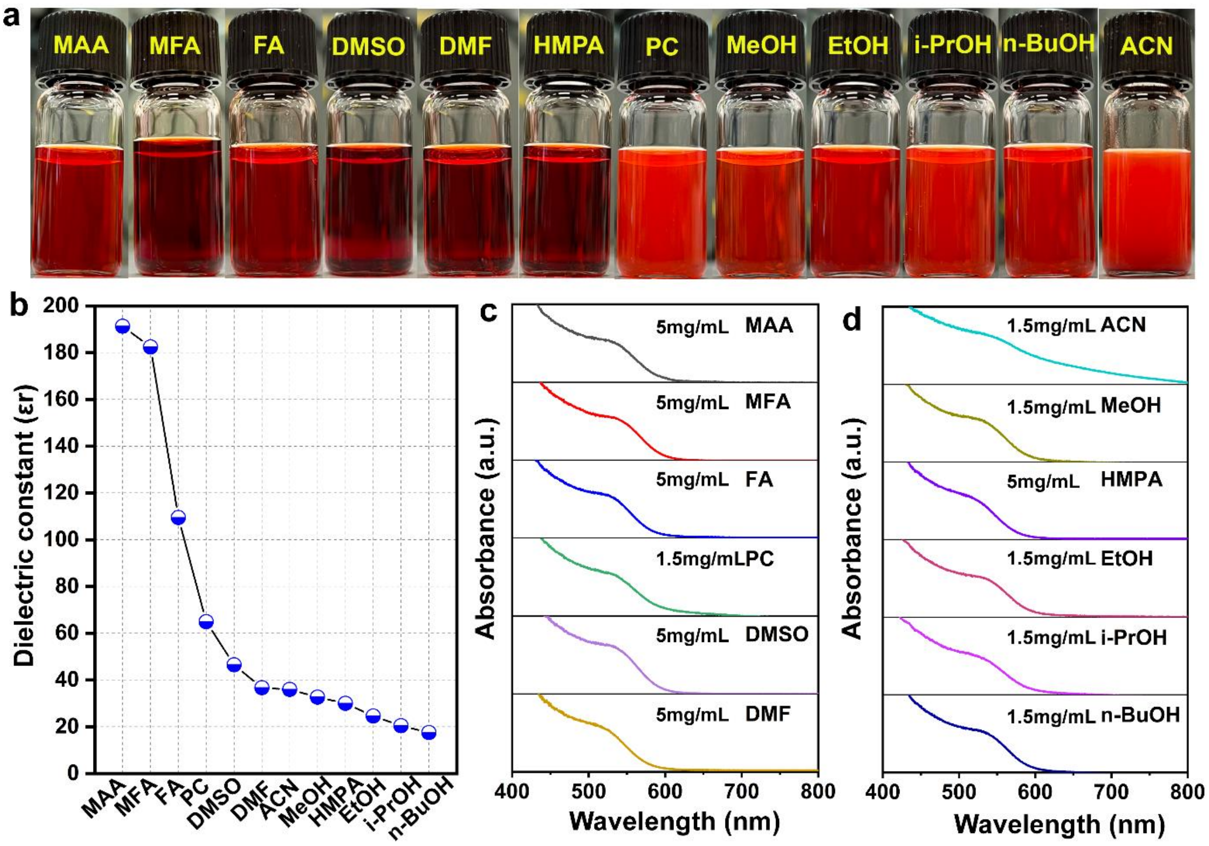

Figure S17. Colloidal dispersity study:(a) photographs of InP QDs after LE with InI_3 salt in different polar solvents, (b) dielectric constant diagram of polar solvents. (c, d) the absorbance spectra of InP QDs after LE with InI_3 salt in different polar solvents.

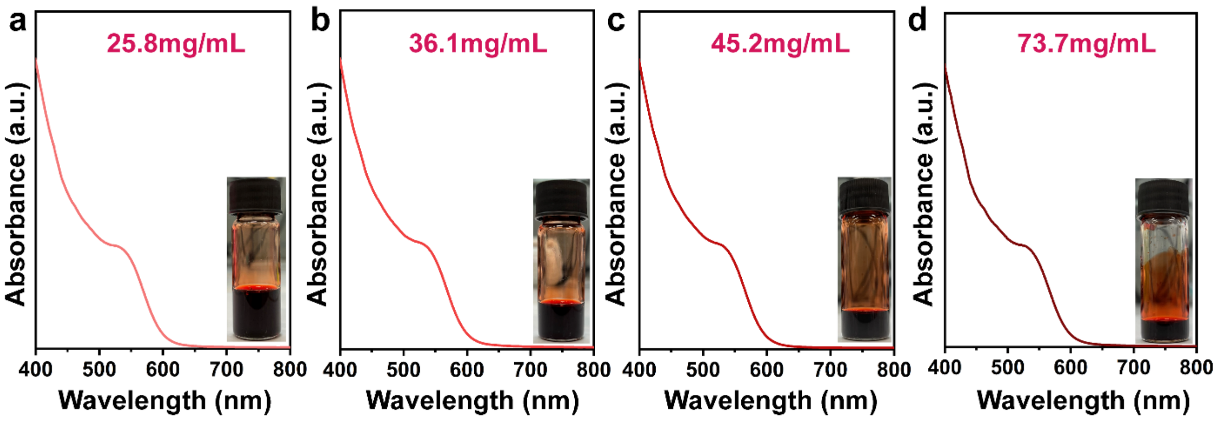

Figure S18. The absorbance spectra of InP/$[In(MFA)_6]^{3+}$ QDs inks in MFA and photographs of inks inserted in corresponding images.

References

(1) Li, Y.; Hou, X.; Dai, X.; Yao, Z.; Lv, L.; Jin, Y.; Peng, X. Stoichiometry-Controlled InP-Based Quantum Dots: Synthesis, Photoluminescence, and Electroluminescence. *J. Am. Chem. Soc.* **2019**, *141* (16), 6448-6452.
(2) Tessier, M. D.; Dupont, D.; De Nolf, K.; De Roo, J.; Hens, Z. Economic and Size-Tunable Synthesis of InP/ZnE (E = S, Se) Colloidal Quantum Dots. *Chem. Mater.* **2015**, *27* (13), 4893-4898.
(3) Ministro, J. A Study on the Synthesis and the Optical Properties of InP-based Quantum Dots. University of Gent, Gent, 2014.
(4) Reiss, P.; Protière, M.; Li, L. Core/Shell Semiconductor Nanocrystals. *Small* **2009**, *5* (2), 154-168.
(5) Calvin, J. J.; Swabeck, J. K.; Sedlak, A. B.; Kim, Y.; Jang, E.; Alivisatos, A. P. Thermodynamic Investigation of Increased Luminescence in Indium Phosphide Quantum Dots by Treatment with Metal Halide Salts. *J. Am. Chem. Soc.* **2020**, *142* (44), 18897-18906.
(6) Peterson, M. D.; Jensen, S. C.; Weinberg, D. J.; Weiss, E. A. Mechanisms for Adsorption of Methyl Viologen on CdS Quantum Dots. *ACS Nano* **2014**, *8* (3), 2826-2837.
(7) Cros-Gagneux, A.; Delpech, F.; Nayral, C.; Cornejo, A.; Coppel, Y.; Chaudret, B. Surface Chemistry of InP Quantum Dots: A Comprehensive Study. *J. Am. Chem. Soc.* **2010**, *132* (51), 18147-18157.
(8) Guzelian, A. A.; Katari, J. E. B.; Kadavanich, A. V.; Banin, U.; Hamad, K.; Juban, E.; Alivisatos, A. P.; Wolters, R. H.; Arnold, C. C.; Heath, J. R. Synthesis of Size-Selected, Surface-Passivated InP Nanocrystals. *J. Phys. Chem.* **1996**, *100* (17), 7212-7219.
(9) M. J. Seong, O. I. M. i., A. J. Nozik, A. Mascarenhas. Size-Dependent Raman Study of InP Quantum Dots. *Appl. Phys. Lett.* **2003**, *82* (2), 185-187.
(10) Salyulev, A. B.; Zakiryanova, I. D. Raman Spectra of Solid, Molten, and Gaseous Gallium Trichloride. *Russ. Metall.* **2010**, *2010* (2), 108-111.
(11) Beattie, I. R.; Gilson, T.; Cocking, P. The Vibrational Spectrum of Ga_2Cl_6. *J. Chem. Soc. A* **1967**, 702-704.
(12) Shamir, J.; Rafaeloff, R. Raman Spectra of Solid Complexes of Trihalides of Antimony and Bismuth with Trihalides of Aluminium and Gallium. *J. Raman Spectrosc.* **1986**, *17* (6), 459-462.
(13) Zhu, G.; Angell, M.; Pan, C.-J.; Lin, M.-C.; Chen, H.; Huang, C.-J.; Lin, J.; Achazi, A. J.; Kaghazchi, P.; Hwang, B.-J.; Dai, H. Rechargeable Aluminum Batteries: Effects of Cations in Ionic Liquid Electrolytes. *RSC Adv.* **2019**, *9* (20), 11322-11330.
(14) Dong, A.; Ye, X.; Chen, J.; Kang, Y. K.; Thomas, G.; James M. Kikkawa; Murray, C. B. A Generalized Ligand-Exchange Strategy Enabling Sequential Surface Functionalization of Colloidal Nanocrystals. *J. Am. Chem. Soc.* **2010**, *133* (4), 998-1006.
(15) Leemans, J.; Dümbgen, K. C.; Minjauw, M. M.; Zhao, Q.; Vantomme, A.; Infante, I.; Detavernier, C.; Hens, Z. Acid–Base Mediated Ligand Exchange on Near-Infrared Absorbing, Indium-Based III–V Colloidal Quantum Dots. *J. Am. Chem. Soc.* **2021**, *143* (11), 4290-4301.
